# Metagenomic evidence of stronger effect of stylo (legume) than bahiagrass (grass) on taxonomic and functional profiles of the soil microbial community

**DOI:** 10.1038/s41598-017-10613-6

**Published:** 2017-08-31

**Authors:** Yang Zhou, Honghui Zhu, Shenglei Fu, Qing Yao

**Affiliations:** 1 College of Horticulture, South China Agricultural University, Guangdong Province Key Laboratory of Microbial Signals and Disease Control, Guangdong Engineering Research Center for Litchi, Guangdong Engineering Research Center for Grass Science, Guangzhou, 510642 China; 2 Guangdong Institute of Microbiology, State Key Laboratory of Applied Microbiology Southern China, Guangdong Provincial Key Laboratory of Microbial Culture Collection and Application, Guangzhou, 510070 China; 3 College of Environment and Planning, Henan University, Kaifeng, 475004 China

## Abstract

Plants are key determinants of soil microbial community (SMC). Legumes and grasses are distinct groups in various ecosystems; however, how they differentially shape SMC structure and functioning has yet to be explored. Here, we investigate SMC in soils grown with stylo (legume) or bahiagrass (grass). Soil metagenomic sequencing indicates that Archaea was more abundant in unplanted soils than in planted soils, and that stylo selected higher abundance of fungi than bahiagrass. When the stylo soils enriched *Streptomyces*, *Frankia*, *Mycobacterium* and *Amycolatopsis*, the bahiagrass soils enriched *Sphingomonas* and *Sphingobium*. NMDS reveals that the legume shaped SMC more greatly than the grass (*P* < 0.004). SMC functional profiles (KEGG and CAZy) were also greatly altered by plants with the legume being more effective (*P* < 0.000 and *P* < 0.000). The abundant microbial taxa contributed to the main community functions, with *Conexibacter*, *Sphingomonas*, and *Burkholderia* showing multifunctionality. Moreover, soil chemical property showed much higher direct effect on SMC structure and functional profiles than soil extracts, although the soil total nitrogen and some compounds (e.g. heptadecane, 1-pentadecyne and nonanoic acid) in soil extracts were best correlated with SMC structure and functional profiles. These findings are the first to suggest that legume species shape SMC more greatly than grass species.

## Introduction

Soil microorganisms and their interactions with plants are of great significance for agricultural production^1^, potentially promoting the crop yield to meet the increasing human requirements for food^2, 3^. Plant-microbial interactions can greatly regulate diverse soil biogeochemical processes, e.g., organic carbon turnover and nitrogen (N) and phosphorus (P) mineralization, which are important determinants of soil fertility and thus of agricultural production^2^. Additionally, plant-microbial interactions have the potential to suppress soil-borne pathogens, enhance stress resistance, and even directly alter plant traits and productivity^4–9^.

It is well-acknowledged that plants can shape the soil microbial community (SMC) structure^10–12^, and the critical role of plants in soil microbial assembly is normally achieved *via* direct and indirect pathways. The direct pathway is through root exudates, the kinds and amounts of which differ substantially depending on the plant species/genotype^13, 14^. Different root exudates support different microbial assemblies in the rhizosphere, with these exudates being directly available foods that serve as nutrient and energy sources^15–17^. The indirect pathway is through different nutrient requirement characteristics and root system architectures, which depend on the plant species/genotype^5^. Soils grown with plants with these differences can vary greatly in their physical and chemical properties, which further support different soil microbial assemblies. Given the essential involvement of soil microorganisms in the biogeochemical processes of nutrients and soil fertility, soil microbial assemblies with different community structures normally vary in their functions^18, 19^. In general, plants can drastically affect belowground ecological processes *via* their regulation of the soil microorganisms in diverse ecosystems.

Legumes and grasses are ubiquitous components of various plant ecosystems and represent a large proportion of agricultural crops as well. It is well-established that legumes and grasses greatly differ in diverse aspects, e.g., a higher content of phenols in the root extracts of legumes than in those of other species^20, 21^ and a higher C:N ratio in the residues of grasses than legumes. The effects of legumes and grasses on soil microbes have been investigated previously but independently, and their differential influences on the SMC structure and functional profiles have been poorly compared and understood. This knowledge gap may greatly weaken our understanding of the biogeochemical processes of the elements in diverse soil ecosystems and further hinder our efforts to improve soil fertility.

In this study, a legume plant, *Stylosanthes guianensis* (SG), and a grass plant, *Paspalum notatum* (PN), were grown in the same agricultural soil to compare the extent to which legumes and grasses can assemble a specific SMC from the community in unplanted soils and shape the SMC functioning. *S*. *guianensis* is a subtropical perennial leguminous plant widely used in orchards as cover crops to increase soil fertility, while *P*. *natatu* is a subtropical perennial gramineae plant commonly used in hilly orchards as cover crops to alleviate soil erosion. As revealed with shotgun metagenomic sequencing, the two plants assembled very different SMCs at both the kingdom and genus levels, which were also both distinct from that in the soil without plants. The Bray-Curtis similarity indicates that stylo altered the SMC structure more greatly than bahiagrass. The two plants also altered the functional profiles (evaluated with KEGG and CAZy), with the legume showing a much stronger effect than the grass. The legume increased the function of metabolism while the grass increased the function of genetic information processing. Linking the taxonomic profiles with functional profiles demonstrates that some genera were specifically responsible for the affected functions. These data suggest a much stronger effect of the legume species on the taxonomic and functional profiles of the SMC than the grass species.

## Results

### Microbial abundance, community structure and plant-specific taxa

**Figure 1:**
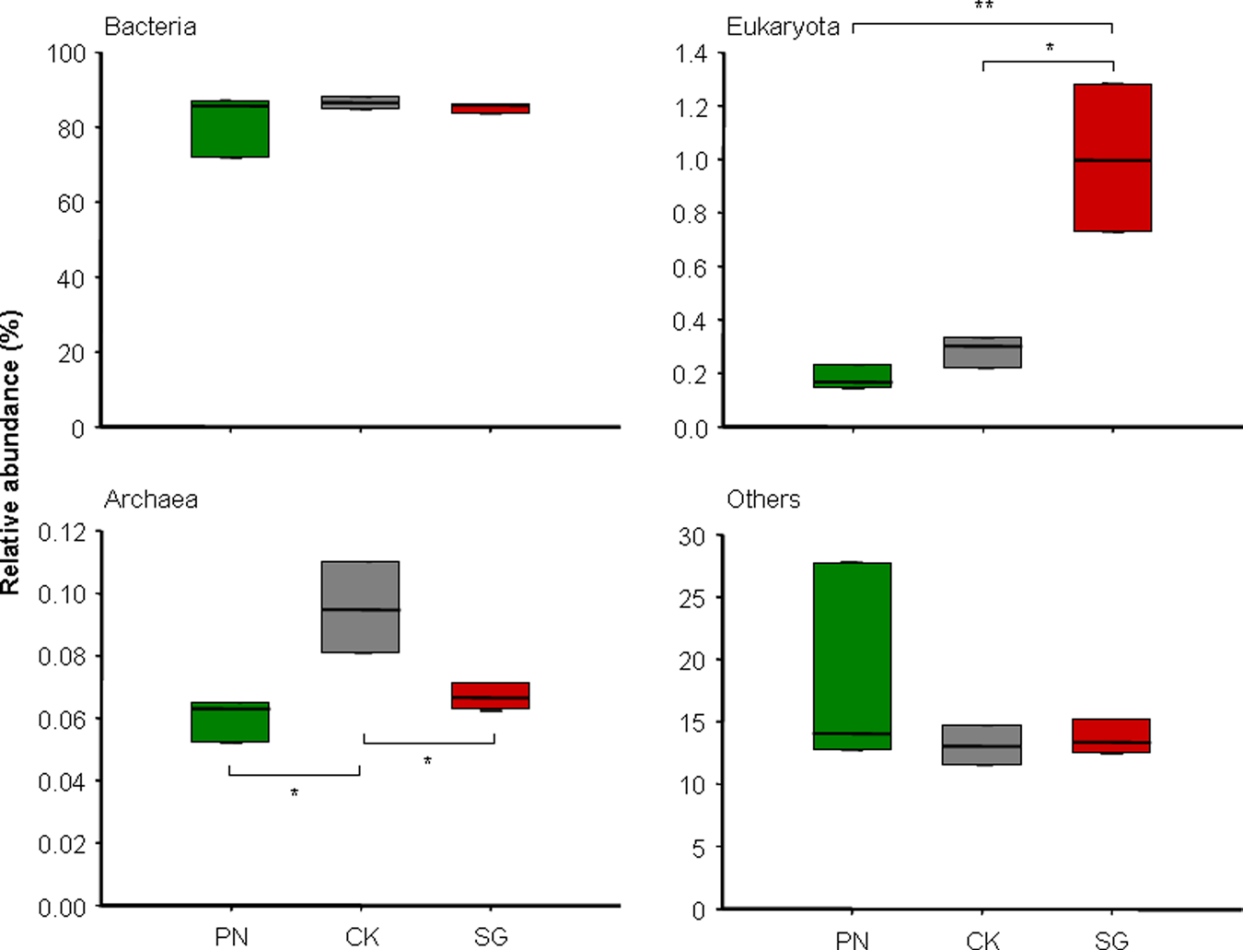
The relative abundance (%) of soil microbial taxa at the kingdom level as regulated by PN and SG. CK, Control; PN, *Paspalum notatum*; SG, *Stylosanthes guianensis*. * and **Indicate significant differences at *P* < 0.05 and *P* < 0.01 (*t*-test), respectively.

Using a shotgun metagenomic approach, we obtained 54.74 Gb of paired-end sequences in total with 48.7 million reads per sample. The evaluations of the raw data and the clean data were shown in Supplementary Table S1. To assess the structure and diversity of the SMC, alignment was performed by BLAST against the micro-NCBI Taxonomy Database extracted from NCBI, and the lowest common ancestor (LCA) algorithm was used with MEGAN. At the kingdom level, the SMCs in the CK, PN and SG soils were similar to one another, with Bacteria being dominant. The relative abundance of Bacteria was 86.52%, 81.57% and 85.26% for the PN, CK and SG soils, respectively, without any significant differences among them (Fig. 1). In contrast, the relative abundance of Eukaryotes was 1.00% in the soil associated with SG, which was significantly higher than in the PN and CK soils (0.18% and 0.29%, respectively) (Fig. 1, ANOVA, *P* < 0.002), indicating a stronger selection for fungi by the legume than grass. For Archaea, both SG and PN significantly decreased the relative abundance (Fig. 1, ANOVA, *P* < 0.009). The quantification of bacterial, archaeal and fungal abundance by using qPCR revealed that the copies of archaeal 16 S rRNA gene were higher but the copies of bacterial 16 S rRNA gene were lower in CK soils than in SG or PN soils, and the highest copies of fungal ITS gene were found in SG soils (Supplementary Table S2). These data confirmed the results of metagenomic data.

**Figure 2:**
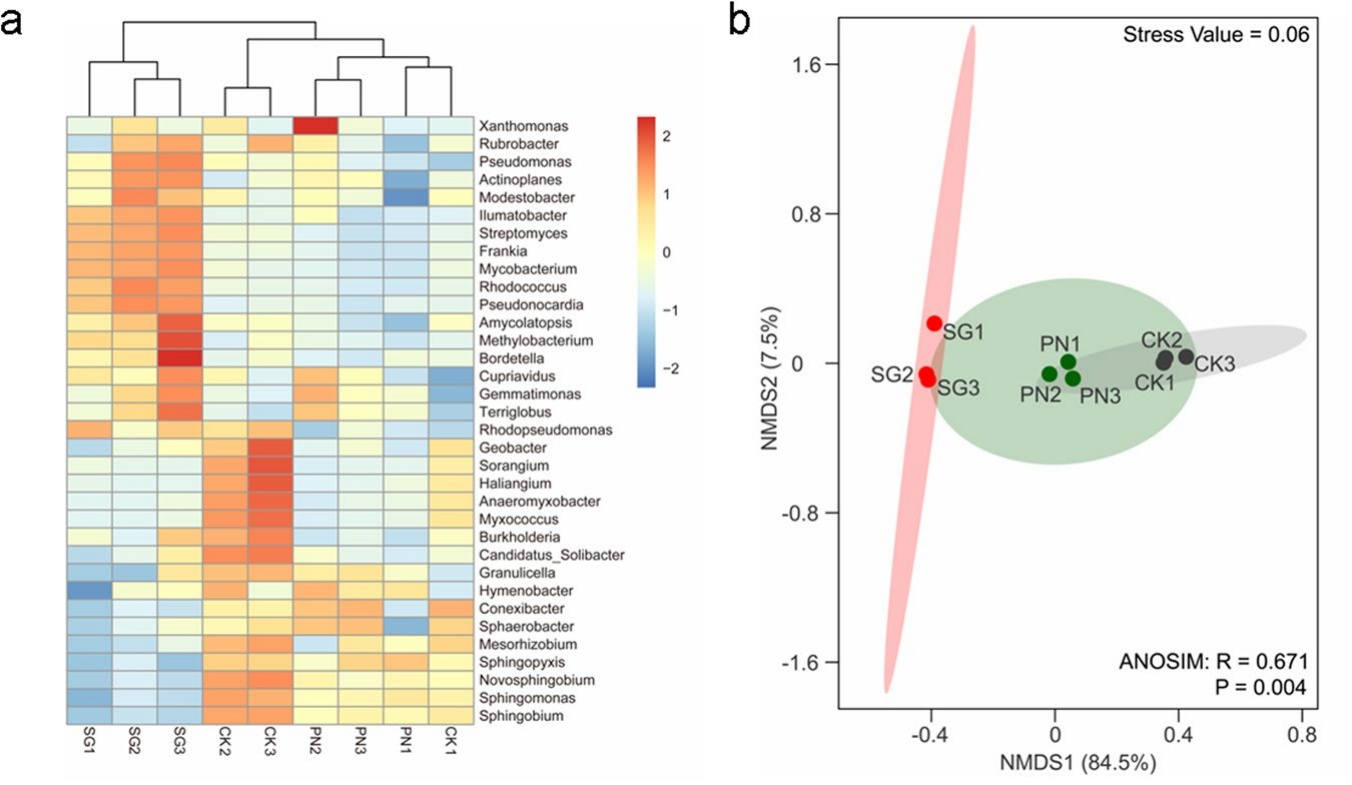
The differential regulation of soil microbial taxa at the genus level (top 30 genera) by PN and SG. CK, Control; PN, *Paspalum notatum*; SG, *Stylosanthes guianensis*. Clustering (**a**) and NMDS (**b**) show a higher similarity of microbial community structure in soils of PN and CK than in soils of SG and CK.

**Figure 3:**
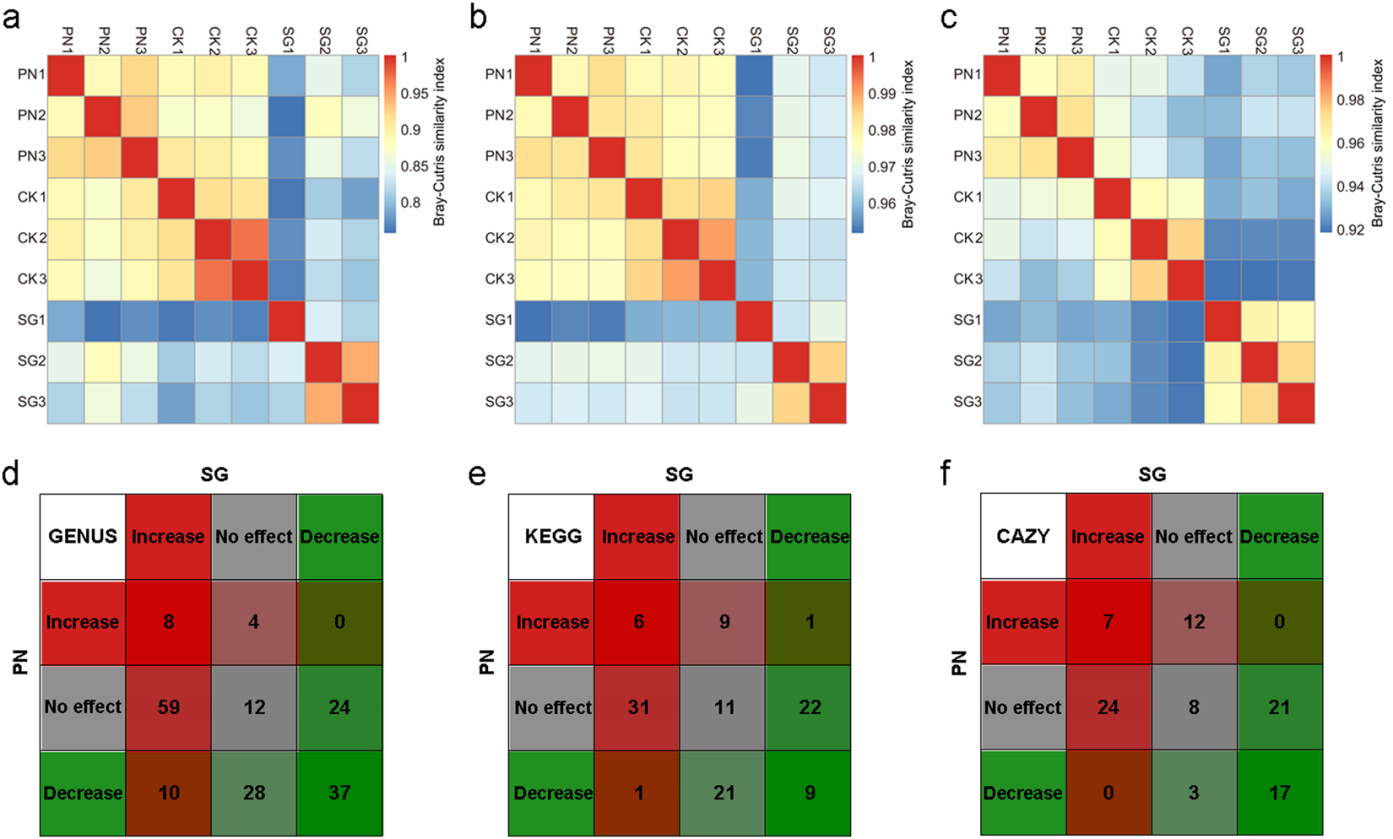
The similarity of soil microbial taxonomic and functional profiles between CK and PN or SG. PN, *Paspalum notatum*; CK, Control; SG, *Stylosanthes guianensis*. (**a**–**c**) the Bray-Curtis similarity index between CK and PN or SG; (**d**–**f**) the number of genera or functions affected by PN or SG. (**a** and **d**) taxonomic profile (genus); (**b** and **e**) KEGG functional profile (level 3); (**c** and **f**) CAZy functional profile (level 2). The similarity coefficient of PN and CK is significantly (*P* = 0.000) higher than that of SG and CK for taxonomic (**a**), KEGG functional (**b**) and CAZy functional (**c**) profiles. “Increase”, “No effect” and “Decrease” indicate a significant increase, no significant change and a significant decrease by PN or SG with comparison to CK, respectively.

At the phylum level, the top 10 most abundant taxa were Proteobacteria (49.86%), Actinobacteria (27.13%), Gemmatimonadetes (17.17%), Acidobacteria (0.84%), Ascomycota (0.50%), Firmicutes (0.47%), Chloroflexi (0.37%), Bacteroidetes (0.36%), Deinococcus-Thermus (0.21%), and Euryarchaeota (0.21%) (Supplementary Data S1). At the genus level, hierarchical clustering revealed that the SMC in the SG soils was much different from that in the CK soil, while the SMC structures in the PN and CK soils grouped together (Fig. 2a), showing the stronger alteration of the SMC structure by SG than by PN. The non-metric multidimensional scaling (NMDS) analysis showed a significant difference (*P* < 0.004) in the SMC structure among the three soils, with the SG soil being more distant from that of CK than that of PN (ANOSIM, R = 0.671, *P* < 0.004, Fig. 2b), supporting the result of hierarchical clustering. Quantitatively, the Bray-Curtis similarity coefficient of the SG and CK generated with the SMC structures was 0.794, which was significantly lower than that for the PN and CK soils (0.883, *P* < 0.000, Fig. 3a, Supplementary Table S3). Taken together, these data strongly suggest that the legume shaped the SMC structure more greatly than grass.

For the abundant genera (relative abundance > 0.1%), the SG soils enriched some taxa, such as *Streptomyces*, *Frankia*, *Mycobacterium* and *Amycolatopsis*; however, the abundance of *Sphingomonas* and *Sphingobium* were higher in PN soils compared with SG soils (Supplementary Data S2). We found that the greater preference for more specific genera by SG than by PN was obvious (77 genera for SG *vs*. 12 genera for PN, Fig. 3d).

### Selection of a potentially functional soil microbial community by plants

To assess the SMC functional profiles, the predicted genes (66138 Open Reading Frames with an average length of 430 bp) were annotated with both the Kyoto Encyclopedia of Genes and Genomes (KEGG) database and the Carbohydrate-Active EnZymes Database (CAZy). In total, 44.00–55.46% sequences were assigned to specific KEGG orthologs and were mainly matched to metabolism, genetic information processing, environmental information processing and cellular processes at level 1.

**Figure 4:**
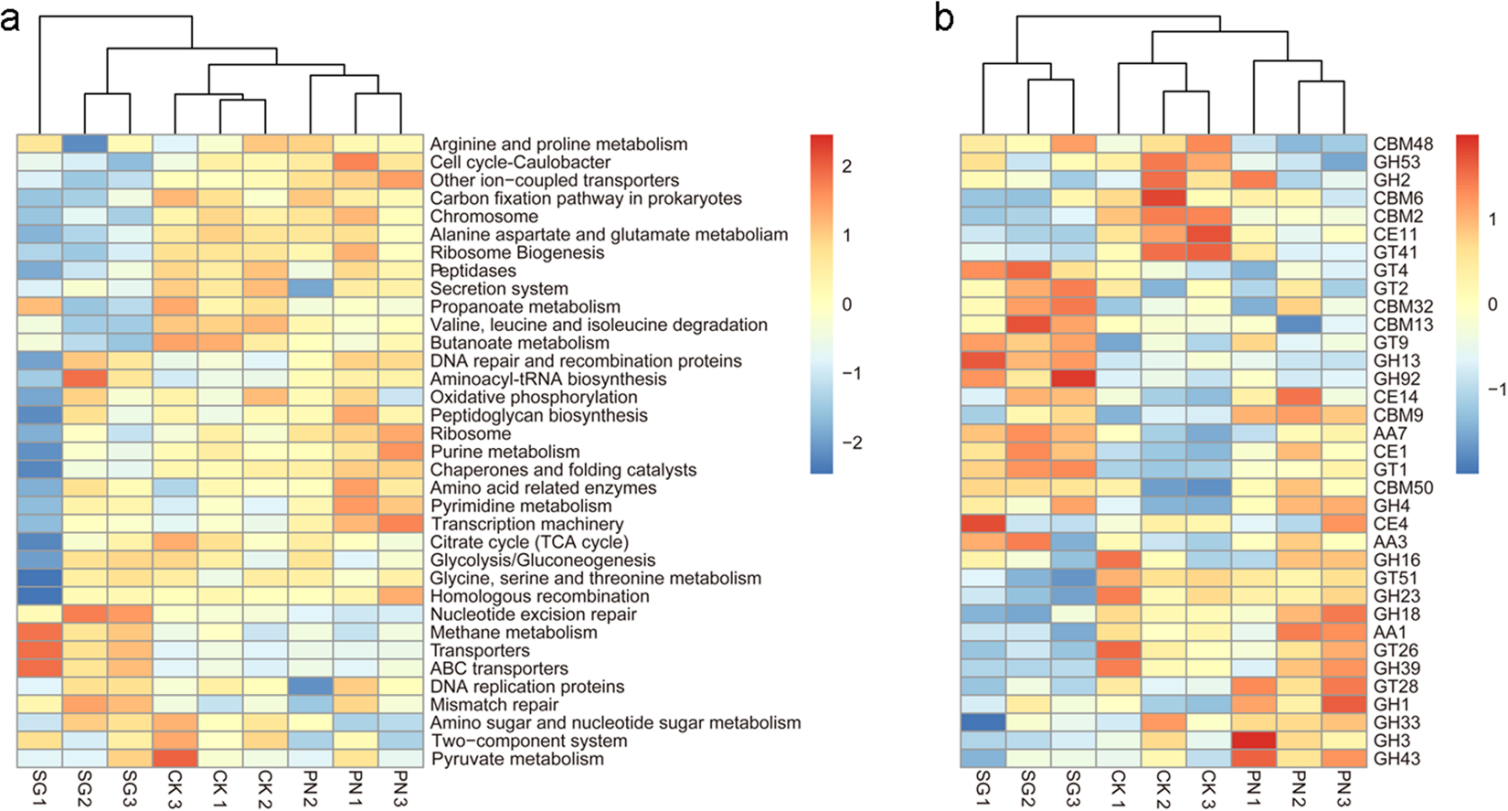
The differential regulation of soil microbial functional profiles by PN and SG. CK, Control; PN, *Paspalum notatum*; SG, *Stylosanthes guianensis*. A higher similarity (refer to Fig. 3b and c) of the functional profiles in the soils of PN and CK than in those of SG and CK is observed for the functional profiles of KEGG level 3 (**a**) and CAZy level 2 (**b**). For each functional profile, only the functions within the top 35 in terms of relative abundance are included.

According to hierarchical clustering on the basis of the SMC functional profiles at KEGG level 3, PN and CK grouped together while SG was separated (Fig. 4a), indicating that the alteration of SMC functional profiles by SG was much greater than that by PN. Similarly, this was also the case for CAZy level 2 (Fig. 4b). Statistically, at KEGG level 3, there were 38 main functions (relative abundance > 0.1%) that were increased by SG, in contrast to only 16 main functions increased by PN (Fig. 3e); at CAZy level 2, 31 main functions (relative abundance > 0.1%) were increased by SG, in contrast to only 19 main functions increased by PN (Fig. 3f). Moreover, the Bray-Curtis similarity coefficients of the SG and CK soils generated with the microbial community functional profiles was 0.964 (KEGG level 3) and 0.923 (CAZy level 2), which were significantly lower than those for the PN and CK soils (0.977 and 0.945, *P* < 0.000 and 0.000, Fig. 3b and c, Supplementary Table S3). These data indicate that, except for the SMC structure, SG altered the SMC functional profiles more greatly than PN.

**Figure 5:**
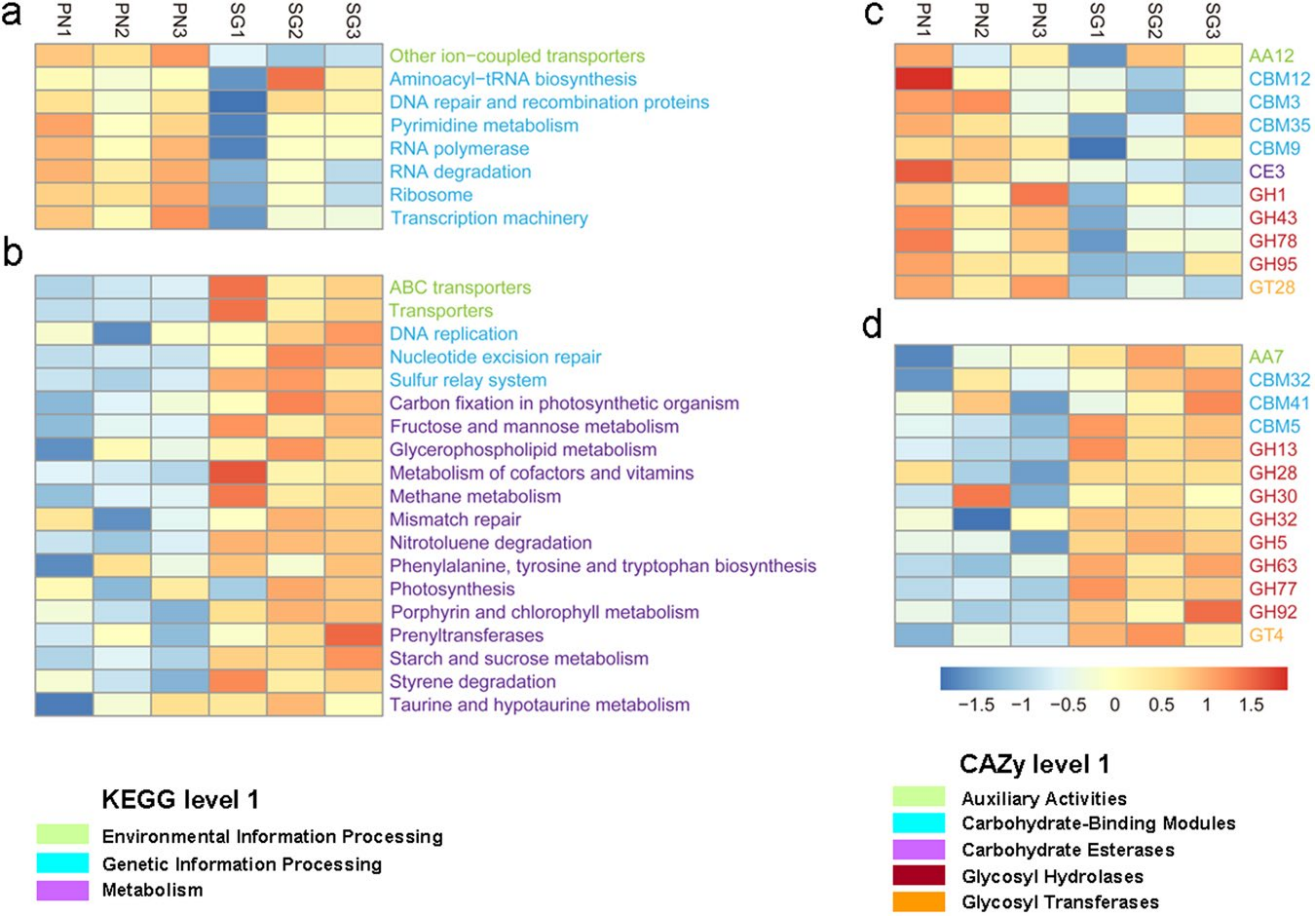
Influence of PN and SG on the soil microbial functional profiles. CK, Control; PN, *Paspalum notatum*; SG, *Stylosanthes guianensis*. (**a** and **b**) KEGG level 3 functions; (**c** and **d**) CAZy level 2 functions. (**a** and **c**) significantly increased by PN; (**b** and **d**) significantly increased by SG. The influence of PN or SG on a specific function was standardized as the fold change in the function with comparison to CK on the basis of the relative abundance.

When evaluated with the fold change in SG/CK and PN/CK, it is clear that SG mainly increased the function of metabolism, while PN mainly increased the function of genetic information processing (KEGG level 1, Fig. 5a and b). For CAZy level 1, SG mainly increased the function of glycosyl hydrolases, while PN mainly increased the functions of carbohydrate-binding modules and glycosyl hydrolases (Fig. 5c and d). These differences highlight the differential regulation of the SMC functional profiles by the legume and the grass.

### Linking community structure to functional profiles

**Figure 6:**
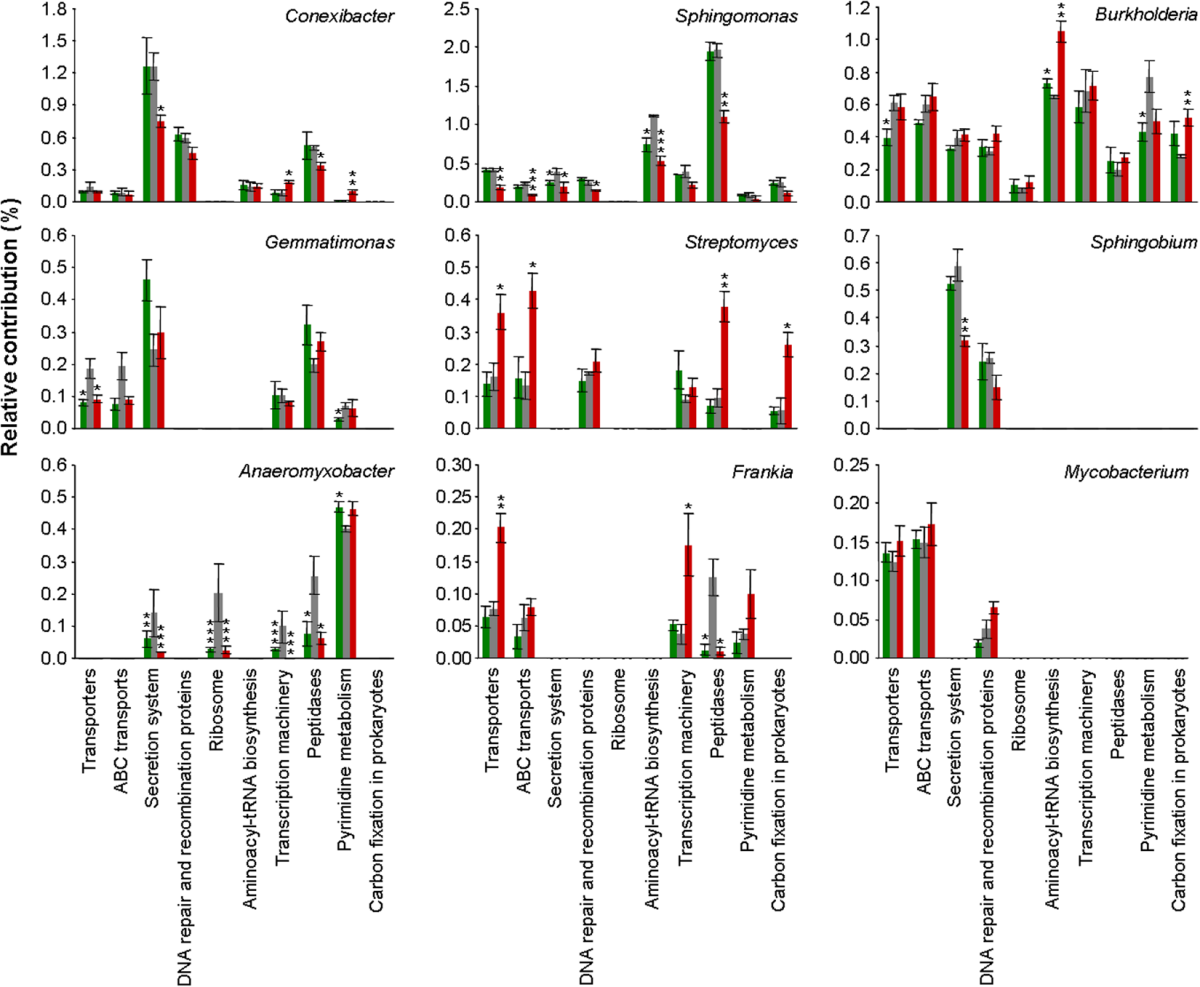
The linkage between the soil microbial taxa and the community functions. PN, *Paspalum notatum*; CK, Control; SG, *Stylosanthes guianensis*. (**a**) the relative contributions of the soil microbial genera (within the top 9 in terms of relative abundance) to the KEGG functions (level 3, within the top 10 in terms of relative abundance). Green, grey and red histograms indicate PN, CK and SG soils. *, ** and *** indicate a significant difference between PN or SG and CK at *P* < 0.05, *P* < 0.01 and *P* < 0.001 (*t*-test), respectively.

To link the SMC structure to the functional profiles, the top 9 genera were evaluated in terms of their contribution to the top 10 specific KEGG level 3 functions. Firstly, among the top 9 genera, 4 genera belong to phylum Proteobacteria (*Sphingomonas*, *Burkholderia*, *Sphingobium*, *Anaeromyxobacter*), 4 to phylum Actinobacteria (*Conexibacter*, *Streptomyces*, *Frankia*, *Mycobacterium*) and 1 to Gemmatimonadetes (*Gemmatimonas*) (Fig. 6). Secondly, *Conexibacter*, *Sphingomonas*, and *Burkholderia* contributed to 8, 9, and 10 of the 10 top functions, respectively, indicating their multifunctionality. In detail, *Conexibacter* mainly contributed to secretion system, with SG significantly decreasing this contribution. *Sphingomonas* mainly contributed to peptidases, with SG significantly decreasing this contribution. *Burkholderia* mainly contributed to aminoacyl-tRNA biosynthesis, with both PN and SG significantly increasing this contribution.

On the other hand, for those abundant genera, it seems that PN promoted the populations of *Sphingomonas* and *Sphingobium* in association with the KEGG level 3 functions of transporters, secretion system, transcription machinery, aminoacyl-tRNA biosynthesis, DNA repair and recombination proteins, peptidases, and carbon fixation pathways in prokaryotes (Table 1). In contrast, SG promoted the populations of *Streptomyces* and *Rhodopseudomonas* in association with the KEGG level 3 functions of transporters, ABC transporters, ribosomes, transcription machinery, and pyrimidine metabolism. Moreover, it is clear that less abundant genera (relative abundance < 0.1%, 16 genera) were not the main taxa contributing to the top 10 functions compared to the abundant genera (34 genera) (Table 1).

**Table 1:** The soil microbial genera with top 5 relative contributions to each of the top 10 KEGG functions. Genera followed by * or # represent significantly higher relative abundance in PN soil or in SG soil, respectively.

KEGG functions		Influence on the specific function		Genera within the top 5 in terms of relative contribution to the specific function
Level 2	Level 3	SG	PN	
Membrane Transport	Transporters	+	○	***Burkholderia***, Sphingomonas*, ***Streptomyces***#, ***Rhodopseudomonas***#, *Pseudonocardia*#
	ABC transporters	+	○	***Burkholderia***, ***Rhodopseudomonas***#, *Pseudonocardia*#, *Sphaerobacter*, ***Streptomyces***#
	Secretion system	+	○	***Conexibacter***, Sphingobium*, ***Pseudomonas, Burkholderia***, ***Gemmatimonas***
Translation	Ribosome	○	+	*Candidatus Saccharimonas*#, ***Anaeromyxobacter***, ***Burkholderia***, *Micromonospora#*, ***Rhodopseudomonas*** *#*
	Transcription machinery	○	+	***Burkholderia***, Sphingomonas*, *Azospirillum*, *Acidobacterium*, ***Streptomyces***#
	Aminoacyl-tRNA biosynthesis	○	+	Burkholderia, Sphingomonas*, *Sulfuricella, Corynebacterium*#, *Acidimicrobium*#
Replication and Repair	DNA repair and recombination proteins	○	+	***Conexibacter***, ***Burkholderia***, *Erythrobacter*, Sphingomonas*, Sphingobium*
Nucleotide Metabolism	Pyrimidine metabolism	○	+	***Burkholderia***, ***Anaeromyxobacter***, ***Bradyrhizobium***, *Hymenobacter*, ***Rhodopseudomonas***#
Enzyme Families	Peptidases	−	○	Sphingomonas*, *Conexibacter, Granulicella*, ***Bradyrhizobium***, ***Gemmatimonas***
Energy Metabolism	Carbon fixation pathways in prokaryotes	−	○	Burkholderia, Sphingomonas*, *Acidimicrobium*#, *Sideroxydans*#, *Leptothrix*#

Genera in bold indicate a relative abundance of > 0.1%. “ + ”, “−”, and “○” indicate significant increase, decrease, and no effect by SG or PN. PN, *Paspalum notatum*; SG, *Stylosanthes guianensis*.

It is noteworthy that some data, if not all, in Fig. 6 and in Table 1 agreed well. For example, Fig. 6 indicates that SG significantly increased the *Streptomyces* population, and *Streptomyces* contributed substantially to the KEGG function of transporters. Consistently, Table 1 shows that SG positively influenced the KEGG function of transporters. This consistency indicates that SG increased the KEGG function of transporters *via* its promotion of the *Streptomyces* population.

### Relationship between environmental cues and microbial community structure/functional profiles

To explore the factors associated with the differential SMC structures and functional profiles, both the compounds in the soil extracts and the soil chemical properties were evaluated. Among the 57 identified compounds, the percentages of 12 compounds were over 1% (Supplementary Data S3), and these were designated as major compounds in the soil extracts. Four major compounds (carbamic acid, benzoic acid, 9,12-octadecadienoic acid, heptadecane) significantly differed in proportion among the three soils, with heptadecane showing the same pattern as those of the SMC structure and functional profiles; namely, SG increased the proportion of heptadecane while PN did not. For the minor compounds, 1-pentadecyne and nonanoic acid also showed a similar pattern. When most soil properties were not affected, the pH, DOC, TN, AK and NO_3_^−^N were significantly different among the soils (Supplementary Table S4), with TN showing the same pattern as those of the SMC structure and functional profiles.

Using the function “bioenv” in the vegan package in R, TN was found to be the most correlated factor among all the soil chemical properties (SCPs) and their combinations. Mantel tests revealed that TN was significantly associated with the SMC structure at the genus level (*P* < 0.003) and the functional profile at KEGG level 3 (*P* < 0.002) (Table 2). Similarly, the combination of nonanoic acid, xylulose, 2-bromo dodecane, 1-pentadecyne, 3-heptadecene, and heptadecane closely related to the SMC structure (*P* < 0.001), while the combination of nonanoic acid, acetamide, cyclohexane, 1-pentadecyne, and heptadecane showed a significant correlation with the SMC functional profile (*P* < 0.001) (Table 2). Taken together, these data suggest that TN, some organic acids and alkanes are key environmental cues involved in the regulation of the SMC structure and functioning by the legume and grass in this study. Moreover, PLS-PM showed that SCP had important direct effects on SMC composition (SMCC, 0.65) and SMC function (SMF, 0.70), and some indirect effects (SMCC, 0.23; SMF, 0.22). In contrast, the direct effects of soil extracts were much smaller (0.28 for both SMCC and SMF) (Supplementary Figure S1).

**Table 2:** Mantel tests of the relationships between the microbial community and environmental cues.

Microbial community	Environmental cues	r	P
Microbial community structure at genus level	nonanoic acid, xylulose, 2-bromo dodecane, 1-pentadecyne, 3-heptadecene, heptadecane	0.940	0.001
	TN	0.710	0.003
Microbial community functional profile at KEGG level 3	nonanoic acid, acetamide, cyclohexane, 1-pentadecyne, heptadecane	0.951	0.001
	TN	0.720	0.002

Environmental cues were categorized into soil chemical properties and soil extracts. Prior to the Mantel test, the most related cues in soil chemical properties or soil extracts were selected using the function “bioenv” in the vegan package in R. TN, total nitrogen.

## Discussion

Plants are essential factors shaping the SMC structure^10, 11^ or even driving the succession of the SMC^22^. In our study, the SMC structure in unplanted soil (CK) was much different from that in planted soils (SG and PN), demonstrating the driving force of plants in the SMC^19, 23^. It is interesting that the abundance of Archaea was significantly reduced by plants as revealed by both metagenomic data and qPCR quantification data, which is the first report of this nature to date. It is well-acknowledged that Archaea normally adapt to harsh environments^24^. In our study, the unplanted soil contained no plant roots and was thus lacking the active organic compounds normally released from roots. This relatively harsh condition compared with the planted soils might lead to a higher abundance of Archaea in unplanted soil.

Different plant species were the forces shaping the SMC both taxonomically and functionally^12, 17, 19, 25^. More specifically, root exudates initiate and modulate the interaction between roots and soil microbes, supporting a specific SMC in the rhizosphere of a particular plant species^16, 26^. It is acknowledged that root exudates account for 5–20% of photosynthates^27^ and that different plant species/genotypes can release distinct profiles of root exudates^28, 29^. Consequently, root exudates can be key determinants of the SMC^26, 30^. The root exudate compositions of legumes and grasses are very different^31, 32^ and therefore likely support different SMCs. In addition, due to different nutrient requirement characteristics, legumes and grasses can provide distinct environmental resources to assemble different SMCs, especially a higher nitrogen level due to symbiosis with rhizobium. In this study, we found that the presence of SG resulted in a higher abundance of fungi than the presence of PN, which is consistent with the results revealed by rRNA metatranscriptomes^12^, in which fungi were highly enriched in the rhizosphere of pea compared with that of wheat or oat. These data suggest a possible preference of legumes for fungi.

At the genus level, the stylo enriched for *Streptomyces*, *Frankia*, *Mycobacterium*, *Amycolatopsis*, and *Rhodopseudomonas* compared with bahiagrass, while the latter enriched *Sphingomonas* and *Sphingobium*. Preferences for some of these taxa by legumes or grasses have been reported previously^12, 33–35^. In a wider context, NMDS reveals that SG was more effective in shaping the SMC structure than PN. Our data, together with previous work, suggest that legumes may possibly shape the SMC structure more greatly than grasses.

Along with the altered SMC structure, the SMC functional profiles were also modified by the presence of SG or PN in this study. The modification of SMC functions by plants has been previously investigated^36, 37^. Knelman *et al*.^37^ demonstrated that differences in the bacterial community composition of vegetated soils contributed to differences in the SMC functions. More importantly, our data demonstrate that SG affected the microbial functional profiles more greatly than PN, which is consistent with its effects on the SMC structure. Recently, the factors determining SMC functional profiles have been addressed^19, 23^. The community composition is considered to be the most important factor^18, 19^; however, edaphic factors also contribute substantially to community functional profiles^38, 39^. We consider that these two viewpoints are not controversial because edaphic factors always act on community functions *via* their effects on community structure.

In this study, SG shaped the SMC structure more greatly than PN, and thus, it is not surprising that SG affected the community functional profiles more greatly than PN as well. In this context, it seems reasonable that legumes affect the SMC functional profiles more greatly than grasses, although information regarding the effects of other legumes on the SMC functional profiles is currently very scarce. It is noteworthy that SG mainly enriched the microbial functions of metabolism in contrast to the microbial functions of genetic information processing enriched by PN. This difference between legumes and grasses has not been reported so far and needs further investigation with more legume and grass species.

This study shows that the abundant taxa mainly contributed to the community functional profiles (in terms of the top 10 KEGG level 3 functions), while the less abundant taxa did not. More interestingly, it seems that these abundant taxa were multifunctional, which is much different from the less abundant taxa. For example, *Burkholderia* (one of the top 5 genera) contributed to 9 out of the top 10 functions, while the least abundant genera contributed to only 1 function. Although it has been demonstrated that rare taxa can contribute to temporal changes in the SMC structure^40^ and molecular functions^41^, most researches demonstrate that abundant taxa are the determinant and that those edaphic factors affecting the populations of the abundant taxa can modify the community functional profiles^38, 41^. In this study, SG and PN supported different abundant taxa and thus led to different community functional profiles.

Our results revealed that the relative abundances of *Streptomyces* and *Sphingomonas* and their contributions to the main functions were much different between PN and SG. Notably, SG enriched *Streptomyces*, many isolated species of which are known as members of plant growth-promoting groups, participants in soil decomposition and providers of plant protection^42–44^. However, *Sphingomonas* was enriched in the PN soil, which agrees with the selection by maize^11^. Haichar *et al*.^16^ claimed that Sphingomonadales are specific to monocotyledons (wheat and maize) and demonstrated their ability to use fresh root exudates from their host plants.

Many environmental cues have been demonstrated to be determinants of SMC structure in diverse soil ecosystems, such as soil pH^45, 46^, soil nutrient levels^47^, soil moisture^48, 49^, soil C:N ratio^50^, and temperature^51^. These facts suggest that the drivers of SMCs can vary greatly depending on the given soil ecosystems with specific characteristics. In our study with a legume and a grass as contrasting hosts, the most related cues of SMC structure were the soil total nitrogen and a combination of compounds in soil extracts, differentiating the effects of the two hosts. Alterations to SMCs by soil nitrogen have been revealed in diverse soils, such as alpine soils^52^ and forest soils^53^. Due to biological nitrogen fixation and the subsequent exudation of organic nitrogen in the legume rhizosphere^20^, the associated soil nitrogen is always increased, as was the case in our study. Root exudates are also regarded as an important factor shaping SMC structure^16^; however, their composition might be modified due to biochemical processes soon after their release into soils. In fact, soil extracts (as examined in our study) are the compounds that directly serve as food and energy for microbes, including root exudates and the hydrolysates of soil organic matter. We found that a combination of compounds in soil extracts (nonanoic acid, xylulose, 2-bromo dodecane, 1-pentadecyne, 3-heptadecene, heptadecane) was mostly associated with the SMC. To our knowledge, most research, if not all, has focused on root exudates, and information on soil extracts is very scarce. Therefore, the roles of these compounds in regulating SMC structure merit deep investigation in the future. However, compared to soil extracts, soil chemical property had a much higher direct effect on microbial composition, suggesting that plant nutrient requirement (especially N by legume *vs* grass) may be a more important mechanism in shaping its associated microbes.

Similarly, the total nitrogen and a different combination of compounds in soil extracts (nonanoic acid, acetamide, cyclohexane, 1-pentadecyne, heptadecane) were closely associated with SMC functioning in our study. The similarity of the environmental cues most related to the SMC structure and functional profiles suggests that environmental cues determine SMC functioning, possibly *via* their effects on the SMC structure, which was hypothesized by Forsberg *et al*.^54^. Again, soil chemical property had more important direct effect on microbial function, possibly reflecting that legumes select the SMC function *via* the modification of soil N more important than root exudate.

With ~55 Gb sequencing data, we conclude that legume species shape the SMC more greatly than grass species. The soil total nitrogen and some compounds (e.g. heptadecane, 1-pentadecyne and nonanoic acid) in soil water extracts were best correlated with SMC structure and functional profiles shaped by plants. After only less than 3 months of growth, the microbiomes of the grass and legume were different with a stronger effect of the latter. Differences in the field are likely to be greater, as the plants typically grown year after year and exhibiting continued selection. Exactly, both pot studies and field experiments are needed and will benefit from greater sequencing depth and more effective bioinformatics tools. Moreover, considering great numbers of species to grass and legume, more representative plants from the two categories should be used to get a more general understanding for their ecological roles in plant-microbial driven belowground processes.

## Materials and Methods

### Original soil description and experimental design

The original soil used in the pot experiment was collected from a vegetable garden located at the Heshan Hilly Land Interdisciplinary Experimental Station of the Chinese Academy Science (HHIES-CAS, E 112° 54′, N 22° 41′) in Guangdong province, with laterite soils and a subtropical monsoon climate. The collected soil samples were air-dried, sieved and chemical analyses were performed. The soil pH was 5.42 with total organic carbon (TOC) and dissolved organic carbon (DOC) contents of 1.04% and 0.03%, respectively. The total nitrogen (TN), phosphorus (TP) and potassium (TK) contents were 0.87, 0.89 and 15.90 g kg^−1^, respectively, and the available nitrogen (AN), phosphorus (AP) and potassium (AK) concentrations were 67.4, 161 and 72.6 mg kg^−1^. The concentrations of nitrate and ammonium were 8.22 and 1.50 mg kg^−1^, respectively. These are considered low for nitrogen and phosphorus and moderate for potassium. The prepared soil in the amount of 4.5 kg was added to each plastic pot (32 cm × 18 cm × 13 cm) for plant growth.

Three treatments were established, including planting with bahiagrass (*Paspalum notatum*, PN, grass), planting with stylo (*Stylosanthes guianensis*, SG, legume), and a control without planting. We selected these two plant species because they are widely grown in subtropical orchards as cover crops to improve soil fertility or to alleviate soil erosion^55^. Each treatment consisted of three replicates. The seeds of PN and SG were germinated in Petri dishes and then grown in plastic pots in a greenhouse. Ten days after planting, the seedlings were thinned to 50 seedlings per pot with similar vigour. The pots were watered every day and weighed every three days to ensure a similar soil moisture of 20%. After 90 days of plant growth, the shoots were cut, and the surface soil layer of approximately 1 cm was discarded to avoid the effects of aboveground disturbance. The roots were carefully removed, and any root debris was picked out with forceps. The soils were homogenized, sieved through a 2 mm mesh, and air dried. To reinforce the rhizosphere effect, these soils were grown with their respective plants for a second season of 70 days.

### Sampling and soil chemical properties

After the second season, the plants and soils were sampled as in the first season. Any visible roots in the soils were removed. Soils were sieved with 2 mm pore sized mesh, and then stored at −80 °C for DNA extraction and GC-MS analysis, or air dried for chemical analysis. The soil pH was determined with deionized water (2.5:1, w/v). The TOC and DOC were analysed by titration after wet oxidation with H_2_SO_4_ and K_2_Cr_2_O_7_^56^. The TN, TP and TK were measured using the Kjeldahl method, the molybdenum blue colorimetric method and the flame photometric method, respectively. The AN, AP (Bray-1 extraction) and AK were measured by the alkali-hydrolysed reduction diffusing method, the colorimetric method and the flame photometric method, respectively. Nitrate and ammonium were extracted using 2 mol l^−1^ calcium chloride and determined quantified by UV spectrophotometry and Nessler’s reagent colorimetry, respectively, in extracted from 2 mol l^−1^ calcium chloride.

### Extraction and identification of compounds in soil extracts with GC-MS

Although root exudates are an important factor regulating the SMC^26, 30^, they are subjected to chemical and biochemical reactions after their release into soils. Apart from root exudates, the hydrolysates of soil organic matter (SOM) can also regulate the SMC. Therefore, soil extracts including the root exudates and their derivatives and SOM hydrolysates were investigated to evaluate their relationships with the SMC structure and its functional profiles.

To extract the compounds from the soils, 6 ml sterile pure water was added to 3 g soil, and 300 μl ribitol (5 mg ml^−1^ in water) was also added as internal standard. The samples were then shaken using ultrasonication for 30 min and then rotated end-over-end for 2 h. After centrifuging at 10 000 g for 10 min, the supernatant was transferred. Three hundred microliters of supernatant was vacuum dried, followed by chemical derivatization. Briefly, oxidation was carried out by dissolving the samples in 100 μl of hydroxylamine (20 mg ml^−1^ in pyridine) and incubating them at 50 °C for 40 min. The samples were further derivatized with the addition of N,O-bis (trimethylsilyl)-trifluoroacetamide (BSTFA) containing 1% trimethylchlorosilane (TMCS) (60 μl) at 70 °C for 1 h to trimethylsilylate. Untargeted GC-MS analysis of the extracts was performed with an Agilent 7890 A gas chromatograph coupled with a 5975 C mass detector. Each sample (1 μl) was injected into the gas chromatograph through an HP-5 capillary column (30 m × 0.32 mm × 0.25 μm). The column temperature was held at 100 °C for 2 min; increased to 184 °C with a temperature gradient of 3 °C min^−1^; increased to 192 °C at 0.5 °C min^−1^ and held for 2 min; and then increased to 280 °C at 10 °C min^−1^ and held for 5 min. Helium (99.999%) was used as a carrier gas with a flow rate at 1.0 ml min^−1^. The mass operating parameters were as follows: the ion source temperature was 200 °C, and the interface temperature was 250 °C. The TIC (total ion current) spectra were recorded in the mass range of 40–1000 atomic mass units (amu) in scanning mode. After the baseline correction and peak matching for the original GC-MS diagram were performed, the peak areas of the metabolic components were calculated automatically by a computerized integrator, and the identification of mass spectra was conducted according to the NIST database (2011). The relative contents of the metabolic components were calculated as the ratio of the peak area for a specific metabolite and the peak area for the internal standard.

### DNA extraction, sequencing, assembly and annotation

The total DNA was extracted from each soil sample using a MoBio PowerSoil DNA extraction kit according to the manufacturer’s protocol. To obtain a sufficient amount of DNA and to avoid bias in the DNA extraction, 3 sub-samples were used for the extraction of DNA, and the obtained DNA was combined to acquire a composite DNA sample. Sequencing libraries were generated using the NEBNext^®^ Ultra^TM^ DNA Library Prep Kit for Illumina (NEB, USA) following the manufacturer’s recommendations and added index codes. Briefly, the DNA sample was fragmented by sonication to a size of 300 bp, and the DNA fragments were then end-polished, A-tailed, and ligated with an adaptor. The constructed libraries were sequenced on an Illumina HiSeq. 2500 platform at the Novogene Bioinformatics Institute, and 125 bp paired-end reads were generated.

The obtained reads were filtered as described by Zhou *et al*.^57^. Briefly, the following types of reads were removed: reads with 40% bases having sequence quality < 5; reads with ≥ 10% unidentified nucleotides; reads with ≥ 15 nt aligned to the adapter. The filtered reads were assembled using the SOAPdenovo2 package^58^ with *k*-mers of 49, 55 and 59, and then selected assembly analysis with the biggest N50 for each sample. Then generated scaftigs (average number of 9441, average length of 694 bp, N50 of 778 bp) were used for gene prediction. Assembled scaftigs were used to conduct ORF detection with MetaGeneMark software^59^. With detected ORFs, CD-HIT software was used to creat a non-redundant ORF sets with a similarity threshold of 95%. Alignment was then performed by BLAST against a micro-NCBI Taxonomy Database extracted from NCBI with a 95% sequence similarity level by the lowest common ancestor algorithm^60^ to ensure biological meaning. BLASTP was used to search the protein sequences of predicted genes with the KEGG database^61^ with an E-value cut-off of 1e^−5^. Searches for carbohydrate-active enzymes were performed using the CAZy database reference dataset found in the CAZymes Analysis Toolkit web service^62^ by protein BLAST with an E-value cut-off of 1e^−5^. The abundance of predicted genes was estimated using a method analogous to the contig coverage in sequence assembly according to Arumugam *et al*.^41^.

### Data analysis

Data analysis was performed in SPSS and R statistical software (version 3.1.0; R Development Core Team, 2014). IBM SPSS statistics software version 21 (SPSS Inc., Chicago, IL) was used to perform analysis of variance (ANOVA). All statistical tests in this study were considered significant at *P* < 0.05. The package pheatmap in R was used to plot the heatmaps. After the function ‘bioenv’ in the vegan package was used to select the optimum environmental factors, the relationships between the soil microbial community and soil chemical properties or compounds in the soil extracts were calculated using the function ‘mantel’ in R with the vegan package. The calculated pairwise Bray-Curtis distances matrix of taxonomic and functional profiles between CK and PN or SG were visualized using the package pheatmap in R. ANOSIM (analysis of similarity) was performed to test whether the differences among the treatments were significantly greater than the differences among the replicates within each treatment in R with the vegan package. Moreover, to understand the direct and indirect effects determining the SMC structure/function caused by soil chemical property and soil exacts, Partial Least Squares Path Model (PLS-PM) were performed for SMC structure and function, respectively. PLS-PM was previously used to explore the direct and indirect effects of environmental factors on SMC functioning^63, 64^. We performed PLS-PM by using the “plspm” package in R.

## Electronic supplementary material

Supplementary information
