# Supplementary material for: Metagenomic evidence of stronger effect of stylo (legume) than bahiagrass (grass) on taxonomic and functional profiles of the soil microbial community
Source: Sci Rep. 2017 Aug 31;7:10195. doi: 10.1038/s41598-017-10613-6 (PMC5579253; doi:10.1038/s41598-017-10613-6)
Supplement: Supplementary file 1 — Supplementary information [file 41598_2017_10613_MOESM1_ESM.doc]

**The title of the paper:**

Metagenomic evidence of stronger effect of stylo (legume) than bahiagrass (grass) on taxonomic and functional profiles of the soil microbial community

**The full names of all the authors and their affiliations:**

Yang Zhou^1^, Honghui Zhu^2,*^, Shenglei Fu^3^, Qing Yao^1,*^

^1^College of Horticulture, South China Agricultural University, Guangdong Province Key Laboratory of Microbial Signals and Disease Control, Guangdong Engineering Research Center for Litchi, Guangdong Engineering Research Center for Grass Science, Guangzhou, 510642, China

^2^ Guangdong Institute of Microbiology, State Key Laboratory of Applied Microbiology Southern China, Guangdong Provincial Key Laboratory of Microbial Culture Collection and Application, Guangzhou, 510070, China

^3^ College of Environment and Planning, Henan University, Kaifeng, 475004, China

^*^ The corresponding authors

**The names, full postal addresses, telephone and fax numbers and e-mail addresses of the corresponding authors:**

Qing Yao, PhD

College of Horticulture

South China Agricultural University

Wushan St. 483, Tianhe Dist., Guangzhou

China, 510642

Tel: 86-20-85280228; Fax: 86-20-85280228; E-mail: yaoqscau@scau.edu.cn

Honghui Zhu, PhD

Guangdong Institute of Microbiology

Xianliezhong Rd. 100, Yuexiu Dist., Guangzhou

China, 510642

Tel: 86-20-87685699; Fax: 86-20-87686803; E-mail: zhuhh@gdim.cn

**Supplementary Figure S1.** Directed graph of the Partial Least Squares Path Model (PLS-PM). a: pathway of soil chemical property, soil extracts and soil microbial community composition; b: pathway of soil chemical property, soil extracts and soil microbial function. Observed (i.e. measured) variables are represented in a rectangular form, while latent variables (i.e. constructs) are represented in an elliptical form. Indicated are the loadings (the correlations between a latent variable and its observed variables), the path coefficients and the coefficients of determination (R^2^) calculated by bootstraps. Models were assessed using the Goodness of Fit statistic, a measure of the overall prediction performance. SCP: soil chemical property; SE, soil extracts; SMCC, soil microbial community composition; SMF, soil microbial community function.


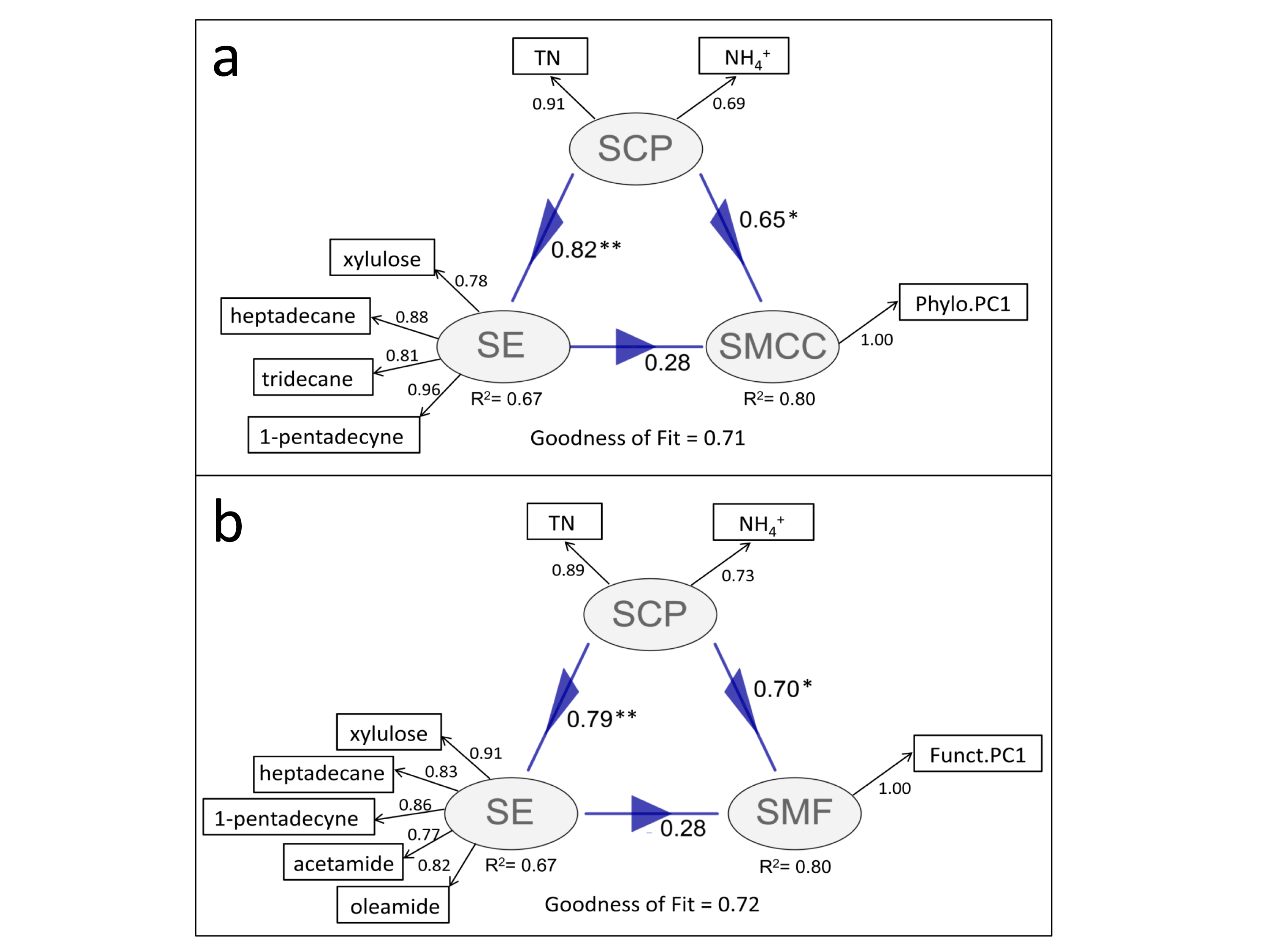


**Supplementary Table S1.** Total nucleotides, number of sequencing reads, clean Q20 and clean Q30 (raw data filtered as described in materials and methods), and the data effectiveness (total bp of raw data/filtered bp of clean data).

| Samples | Total bp (Gb) | No. of reads | Clean Q20 | Clean Q30 | Effective (%) |
| --- | --- | --- | --- | --- | --- |
| PN1 | 6.33 | 50,606,474 | 93.09 | 86.17 | 93.93 |
| PN2 | 5.94 | 47,492,730 | 93.53 | 86.77 | 95.54 |
| PN3 | 6.24 | 49,922,470 | 92.16 | 84.66 | 92.43 |
| SG1 | 6.17 | 49,372,918 | 92.04 | 84.30 | 92.59 |
| SG2 | 6.26 | 50,083,714 | 93.69 | 87.27 | 94.42 |
| SG3 | 5.11 | 40,867,692 | 94.06 | 87.86 | 94.92 |
| CK1 | 6.12 | 48,925,192 | 94.09 | 88.01 | 95.00 |
| CK2 | 6.35 | 50,839,526 | 93.94 | 87.65 | 94.84 |
| CK3 | 6.22 | 49,764,452 | 93.80 | 87.38 | 94.39 |

**Supplementary Table S2.** The abundance of bacterial and archaea 16S rRNA, and fungal ITS genes in soils. CK; Control; PN: *Paspalum natatum*; SG: *Stylosanthes guianensis*. Different letters in each column indicate the significant difference between treatments (Duncan’s multiple range test, *P*<0.05).

| Treatment | Bacteria  (×109 copies/g soil) | Fungi  (×108 copies/g soil) | Archaea  (×107 copies/g soil) |
| --- | --- | --- | --- |
| PN | 6.96±0.52 b | 4.74±0.29 b | 1.60±0.22 a |
| SG | 7.66±0.40 b | 7.96±1.48 c | 1.41±0.12 a |
| CK | 1.26±0.46 a | 1.22±0.16 a | 3.23±0.62 b |

**Supplementary Table S3.** The similarity analysis (Bray-Cutis) between CK and PN or SG for the microbial community structure and functional profiles. The community structure was based on the genus level. The functional profiles included KEGG level 3 and CAZy level 2. CK; Control; PN: *Paspalum natatum*; SG: *Stylosanthes guianensis*. The *t* test was performed on the similarity coefficients between CK *vs* PN and CK *vs* SG.

| Pairs | Microbial community structure | Microbial community functional profiles | |
| --- | --- | --- | --- |
|  |  | KEGG | CAZy |
| CK *vs* PN | 0.883±0.005 | 0.977±0.001 | 0.945±0.003 |
| CK *vs* SG | 0.794±0.009 | 0.964±0.001 | 0.923±0.002 |
| *P* value | 0.000 | 0.000 | 0.000 |

**Supplementary Table S4.** The soil chemical properties as affected by PN and SG. CK, Control; PN, *Paspalum* *natatum*; SG, *Stylosanthes* *guianensis*. TOC, total organic carbon; DOC, dissolved organic carbon; TN, total nitrogen; TP, total phosphorus; TK, total potassium; AN, available nitrogen; AP, available phosphorus (Bray-1 extraction); AK, available potassium. Different letters in each column indicate the significant difference between treatments (Duncan’s multiple range test, *P*<0.05).

| Soil chemical property | PN | CK | SG |
| --- | --- | --- | --- |
| pH | 5.82±0.15 a | 5.25±0.01 b | 5.47±0.03 b |
| TOC (g kg^-1^) | 9.65±0.44 a | 8.86±0.65 a | 10.33±0.32 a |
| DOC (mg kg^-1^) | 203.1±8.3 a | 145.1±8.3 b | 170.0±21.9 ab |
| TN (g kg^-1^) | 0.65±0.02 b | 0.63±0.02 b | 0.74±0.01 a |
| TP (g kg^-1^) | 0.73±0.01 a | 0.64±0.02 a | 0.69±0.03 a |
| TK (g kg^-1^) | 13.13±0.41 a | 12.40±0.31 a | 12.97±0.59 a |
| AN (mg kg^-1^) | 65.4±4.4 a | 58.2±3.2 a | 68.9±5.3 a |
| AP (mg kg^-1^) | 158.0±1.5 a | 167.7±3.2 a | 164.7±3.3 a |
| AK (mg kg^-1^) | 13.4±0.8 b | 68.7±1.4 a | 15.4±0.3 b |
| C:N | 14.8±0.7 a | 14.1±1.4 a | 14.0±0.5 a |
| NO^3-^-N (mg kg^-1^) | 5.57±0.49 b | 19.30±1.72 a | 4.97±0.15 b |
| NH^4+^-N (mg kg^-1^) | 1.09±0.03 a | 0.89±0.05 a | 0.87±0.16 a |

**Supplementary Data S1.** Relative abundance (%) of soil microbial community composition at phylum level. CK; Control; PN: *Paspalum natatum*; SG: *Stylosanthes guianensis*.

| Taxonomy | PN1 | PN2 | PN3 | SG1 | SG2 | SG3 | CK1 | CK2 | CK3 | Total average |
| --- | --- | --- | --- | --- | --- | --- | --- | --- | --- | --- |
| Proteobacteria | 54.638 | 45.361 | 50.068 | 56.951 | 40.948 | 40.406 | 50.716 | 54.587 | 55.099 | 49.864 |
| Actinobacteria | 20.869 | 27.885 | 25.585 | 25.423 | 33.823 | 33.003 | 29.190 | 24.203 | 24.149 | 27.125 |
| Gemmatimonadetes | 18.814 | 20.764 | 18.430 | 13.005 | 18.582 | 19.691 | 14.405 | 15.633 | 15.248 | 17.175 |
| Unclassified | 2.177 | 2.221 | 2.296 | 1.835 | 2.564 | 2.546 | 2.146 | 2.050 | 1.997 | 2.204 |
| Acidobacteria | 0.878 | 1.007 | 0.935 | 0.589 | 0.838 | 1.036 | 0.683 | 0.779 | 0.802 | 0.838 |
| Ascomycota | 0.450 | 0.237 | 0.215 | 0.323 | 0.596 | 0.826 | 0.758 | 0.647 | 0.471 | 0.502 |
| Firmicutes | 0.450 | 0.423 | 0.517 | 0.385 | 0.611 | 0.546 | 0.427 | 0.355 | 0.394 | 0.456 |
| Chloroflexi | 0.354 | 0.422 | 0.408 | 0.275 | 0.359 | 0.353 | 0.392 | 0.360 | 0.387 | 0.368 |
| Bacteroidetes | 0.376 | 0.371 | 0.342 | 0.282 | 0.372 | 0.388 | 0.319 | 0.395 | 0.378 | 0.358 |
| Deinococcus-Thermus | 0.219 | 0.215 | 0.221 | 0.164 | 0.252 | 0.259 | 0.192 | 0.189 | 0.223 | 0.215 |
| Euryarchaeota | 0.173 | 0.197 | 0.193 | 0.149 | 0.188 | 0.192 | 0.237 | 0.257 | 0.295 | 0.209 |
| Cyanobacteria | 0.200 | 0.229 | 0.276 | 0.168 | 0.302 | 0.248 | 0.147 | 0.144 | 0.135 | 0.205 |
| Planctomycetes | 0.057 | 0.058 | 0.081 | 0.059 | 0.078 | 0.094 | 0.095 | 0.093 | 0.112 | 0.081 |
| Verrucomicrobia | 0.077 | 0.067 | 0.075 | 0.073 | 0.085 | 0.071 | 0.081 | 0.095 | 0.094 | 0.080 |
| Chlorobi | 0.040 | 0.044 | 0.046 | 0.062 | 0.073 | 0.069 | 0.031 | 0.033 | 0.030 | 0.048 |
| Candidatus Saccharibacteria | 0.033 | 0.019 | 0.038 | 0.080 | 0.101 | 0.085 | 0.019 | 0.021 | 0.014 | 0.046 |
| Tenericutes | 0.000 | 0.309 | 0.038 | 0.000 | 0.000 | 0.000 | 0.000 | 0.000 | 0.000 | 0.039 |
| Nitrospirae | 0.029 | 0.024 | 0.048 | 0.017 | 0.032 | 0.035 | 0.055 | 0.036 | 0.037 | 0.035 |
| Ignavibacteriae | 0.035 | 0.040 | 0.046 | 0.019 | 0.031 | 0.030 | 0.031 | 0.023 | 0.026 | 0.031 |
| Spirochaetes | 0.029 | 0.036 | 0.041 | 0.019 | 0.019 | 0.022 | 0.021 | 0.034 | 0.036 | 0.029 |
| Basidiomycota | 0.003 | 0.001 | 0.003 | 0.068 | 0.080 | 0.042 | 0.011 | 0.019 | 0.022 | 0.028 |
| Chrysiogenetes | 0.024 | 0.025 | 0.027 | 0.019 | 0.026 | 0.021 | 0.013 | 0.017 | 0.013 | 0.020 |
| Glomeromycota | 0.023 | 0.019 | 0.039 | 0.015 | 0.022 | 0.016 | 0.000 | 0.000 | 0.000 | 0.015 |
| Armatimonadetes | 0.022 | 0.013 | 0.013 | 0.004 | 0.010 | 0.013 | 0.007 | 0.006 | 0.005 | 0.010 |
| Unclassified | 0.010 | 0.007 | 0.007 | 0.015 | 0.005 | 0.008 | 0.004 | 0.004 | 0.010 | 0.008 |
| Unclassified | 0.017 | 0.007 | 0.014 | 0.000 | 0.000 | 0.000 | 0.000 | 0.000 | 0.001 | 0.004 |
| Thermodesulfobacteria | 0.000 | 0.000 | 0.000 | 0.000 | 0.002 | 0.000 | 0.009 | 0.009 | 0.006 | 0.003 |
| Thaumarchaeota | 0.001 | 0.000 | 0.000 | 0.001 | 0.000 | 0.001 | 0.007 | 0.006 | 0.009 | 0.003 |
| Acetothermia | 0.001 | 0.000 | 0.000 | 0.001 | 0.000 | 0.000 | 0.006 | 0.004 | 0.006 | 0.002 |

**Supplementary Data S2.** Relative abundance (%) of soil microbial community composition at genus level. CK; Control; PN: *Paspalum natatum*; SG: *Stylosanthes guianensis*.

| Taxonomy | PN1 | PN2 | PN3 | SG1 | SG2 | SG3 | CK1 | CK2 | CK3 | Total average |
| --- | --- | --- | --- | --- | --- | --- | --- | --- | --- | --- |
| *Conexibacter* | 0.943 | 1.497 | 1.534 | 0.829 | 0.993 | 0.923 | 1.554 | 1.310 | 1.303 | 1.210 |
| *Sphingomonas* | 0.800 | 0.719 | 0.751 | 0.482 | 0.595 | 0.559 | 0.766 | 0.920 | 0.899 | 0.721 |
| *Bradyrhizobium* | 0.181 | 0.201 | 0.263 | 2.842 | 0.209 | 0.240 | 0.227 | 0.216 | 0.221 | 0.511 |
| *Burkholderia* | 0.358 | 0.365 | 0.388 | 0.410 | 0.375 | 0.490 | 0.418 | 0.504 | 0.531 | 0.426 |
| *Gemmatimonas* | 0.327 | 0.396 | 0.338 | 0.320 | 0.378 | 0.411 | 0.264 | 0.317 | 0.302 | 0.339 |
| *Streptomyces* | 0.175 | 0.200 | 0.164 | 0.411 | 0.436 | 0.462 | 0.216 | 0.246 | 0.245 | 0.284 |
| *Sphingobium* | 0.216 | 0.210 | 0.223 | 0.147 | 0.164 | 0.156 | 0.231 | 0.266 | 0.269 | 0.209 |
| *Anaeromyxobacter* | 0.119 | 0.077 | 0.114 | 0.099 | 0.098 | 0.128 | 0.240 | 0.337 | 0.397 | 0.179 |
| *Frankia* | 0.076 | 0.110 | 0.072 | 0.296 | 0.315 | 0.321 | 0.126 | 0.129 | 0.127 | 0.175 |
| *Mycobacterium* | 0.082 | 0.105 | 0.086 | 0.238 | 0.248 | 0.265 | 0.121 | 0.131 | 0.112 | 0.154 |
| *Pseudomonas* | 0.117 | 0.143 | 0.123 | 0.140 | 0.173 | 0.175 | 0.108 | 0.140 | 0.132 | 0.139 |
| *Sorangium* | 0.092 | 0.083 | 0.092 | 0.103 | 0.095 | 0.094 | 0.145 | 0.193 | 0.230 | 0.125 |
| *Actinoplanes* | 0.091 | 0.127 | 0.123 | 0.125 | 0.149 | 0.150 | 0.116 | 0.106 | 0.118 | 0.123 |
| *Amycolatopsis* | 0.077 | 0.101 | 0.089 | 0.122 | 0.139 | 0.160 | 0.111 | 0.111 | 0.111 | 0.114 |
| *Geobacter* | 0.086 | 0.094 | 0.104 | 0.080 | 0.098 | 0.110 | 0.128 | 0.137 | 0.163 | 0.111 |
| *Rhodopseudomonas* | 0.097 | 0.093 | 0.102 | 0.117 | 0.104 | 0.115 | 0.094 | 0.112 | 0.116 | 0.105 |
| *Ilumatobacter* | 0.038 | 0.096 | 0.027 | 0.160 | 0.178 | 0.190 | 0.049 | 0.057 | 0.056 | 0.095 |
| *Sphingopyxis* | 0.103 | 0.084 | 0.101 | 0.064 | 0.074 | 0.064 | 0.090 | 0.101 | 0.101 | 0.087 |
| *Sphaerobacter* | 0.062 | 0.096 | 0.096 | 0.067 | 0.074 | 0.080 | 0.094 | 0.085 | 0.092 | 0.083 |
| *Methylobacterium* | 0.065 | 0.072 | 0.070 | 0.092 | 0.091 | 0.110 | 0.070 | 0.068 | 0.078 | 0.079 |
| *Mesorhizobium* | 0.075 | 0.064 | 0.082 | 0.059 | 0.063 | 0.069 | 0.086 | 0.089 | 0.092 | 0.075 |
| *Novosphingobium* | 0.075 | 0.076 | 0.069 | 0.047 | 0.057 | 0.051 | 0.073 | 0.095 | 0.099 | 0.071 |
| *Cupriavidus* | 0.061 | 0.080 | 0.071 | 0.075 | 0.071 | 0.084 | 0.055 | 0.072 | 0.064 | 0.070 |
| *Myxococcus* | 0.043 | 0.029 | 0.037 | 0.036 | 0.035 | 0.044 | 0.095 | 0.135 | 0.153 | 0.068 |
| *Terriglobus* | 0.061 | 0.079 | 0.063 | 0.059 | 0.076 | 0.090 | 0.045 | 0.055 | 0.049 | 0.064 |
| *Rubrobacter* | 0.048 | 0.063 | 0.055 | 0.051 | 0.068 | 0.071 | 0.058 | 0.057 | 0.070 | 0.060 |
| *Rhodococcus* | 0.032 | 0.041 | 0.033 | 0.089 | 0.107 | 0.101 | 0.042 | 0.046 | 0.042 | 0.059 |
| *Bordetella* | 0.055 | 0.052 | 0.051 | 0.060 | 0.064 | 0.078 | 0.054 | 0.053 | 0.056 | 0.058 |
| *Pseudonocardia* | 0.030 | 0.034 | 0.018 | 0.096 | 0.117 | 0.114 | 0.031 | 0.027 | 0.034 | 0.056 |
| *Modestobacter* | 0.037 | 0.055 | 0.051 | 0.054 | 0.067 | 0.063 | 0.055 | 0.056 | 0.049 | 0.054 |
| *Xanthomonas* | 0.046 | 0.067 | 0.049 | 0.048 | 0.055 | 0.048 | 0.047 | 0.055 | 0.047 | 0.051 |
| *Candidatus* *Solibacter* | 0.041 | 0.049 | 0.044 | 0.038 | 0.044 | 0.056 | 0.048 | 0.069 | 0.070 | 0.051 |
| *Granulicella* | 0.049 | 0.054 | 0.056 | 0.039 | 0.039 | 0.055 | 0.043 | 0.059 | 0.060 | 0.051 |
| *Haliangium* | 0.033 | 0.021 | 0.026 | 0.027 | 0.024 | 0.026 | 0.064 | 0.090 | 0.111 | 0.047 |
| *Hymenobacter* | 0.047 | 0.050 | 0.047 | 0.034 | 0.043 | 0.044 | 0.040 | 0.050 | 0.042 | 0.044 |
| *Caulobacter* | 0.046 | 0.044 | 0.040 | 0.034 | 0.035 | 0.041 | 0.046 | 0.053 | 0.049 | 0.043 |
| *Sinorhizobium* | 0.039 | 0.037 | 0.049 | 0.052 | 0.040 | 0.043 | 0.039 | 0.035 | 0.036 | 0.041 |
| *Phenylobacterium* | 0.041 | 0.044 | 0.036 | 0.040 | 0.036 | 0.039 | 0.043 | 0.047 | 0.044 | 0.041 |
| *Stigmatella* | 0.031 | 0.032 | 0.031 | 0.026 | 0.028 | 0.041 | 0.038 | 0.059 | 0.064 | 0.039 |
| *Deinococcus* | 0.037 | 0.038 | 0.037 | 0.030 | 0.039 | 0.049 | 0.032 | 0.036 | 0.040 | 0.038 |
| *Rhizobium* | 0.031 | 0.032 | 0.036 | 0.036 | 0.036 | 0.031 | 0.039 | 0.038 | 0.043 | 0.036 |
| *Cellulomonas* | 0.024 | 0.022 | 0.017 | 0.041 | 0.042 | 0.044 | 0.035 | 0.040 | 0.048 | 0.035 |
| *Azospirillum* | 0.032 | 0.025 | 0.025 | 0.031 | 0.033 | 0.043 | 0.032 | 0.041 | 0.045 | 0.034 |
| *Nocardia* | 0.027 | 0.024 | 0.018 | 0.050 | 0.049 | 0.051 | 0.027 | 0.029 | 0.030 | 0.034 |
| *Candidatus Koribacter* | 0.026 | 0.039 | 0.035 | 0.030 | 0.035 | 0.046 | 0.023 | 0.020 | 0.023 | 0.031 |
| *Catenulispora* | 0.019 | 0.023 | 0.026 | 0.028 | 0.034 | 0.030 | 0.035 | 0.032 | 0.035 | 0.029 |
| *Rhodanobacter* | 0.025 | 0.026 | 0.029 | 0.024 | 0.033 | 0.035 | 0.025 | 0.034 | 0.031 | 0.029 |
| *Variovorax* | 0.024 | 0.023 | 0.019 | 0.035 | 0.033 | 0.038 | 0.023 | 0.030 | 0.029 | 0.028 |
| *Streptosporangium* | 0.017 | 0.024 | 0.017 | 0.042 | 0.041 | 0.040 | 0.022 | 0.021 | 0.027 | 0.028 |
| *Clavibacter* | 0.020 | 0.028 | 0.034 | 0.021 | 0.019 | 0.020 | 0.034 | 0.036 | 0.033 | 0.027 |
| *Desulfatibacillum* | 0.021 | 0.037 | 0.034 | 0.017 | 0.023 | 0.021 | 0.036 | 0.028 | 0.027 | 0.027 |
| *Blastococcus* | 0.020 | 0.024 | 0.021 | 0.032 | 0.027 | 0.039 | 0.031 | 0.028 | 0.022 | 0.027 |
| *Dyella* | 0.027 | 0.021 | 0.026 | 0.025 | 0.027 | 0.030 | 0.026 | 0.029 | 0.024 | 0.026 |
| *Symbiobacterium* | 0.023 | 0.027 | 0.028 | 0.024 | 0.030 | 0.033 | 0.022 | 0.022 | 0.025 | 0.026 |
| *Nocardiopsis* | 0.024 | 0.023 | 0.028 | 0.019 | 0.025 | 0.026 | 0.026 | 0.027 | 0.034 | 0.026 |
| *Roseiflexus* | 0.026 | 0.026 | 0.022 | 0.024 | 0.030 | 0.030 | 0.021 | 0.024 | 0.026 | 0.025 |
| *Erythrobacter* | 0.024 | 0.021 | 0.026 | 0.022 | 0.021 | 0.020 | 0.029 | 0.028 | 0.032 | 0.025 |
| *Azoarcus* | 0.016 | 0.023 | 0.023 | 0.020 | 0.018 | 0.019 | 0.030 | 0.033 | 0.040 | 0.025 |
| *Nakamurella* | 0.009 | 0.014 | 0.008 | 0.041 | 0.052 | 0.056 | 0.013 | 0.011 | 0.014 | 0.024 |
| *Kribbella* | 0.008 | 0.015 | 0.008 | 0.041 | 0.042 | 0.050 | 0.016 | 0.017 | 0.020 | 0.024 |
| *Cyanobium* | 0.020 | 0.029 | 0.028 | 0.019 | 0.033 | 0.028 | 0.017 | 0.020 | 0.022 | 0.024 |
| *Micromonospora* | 0.012 | 0.018 | 0.012 | 0.035 | 0.046 | 0.041 | 0.015 | 0.019 | 0.015 | 0.024 |
| *Ralstonia* | 0.023 | 0.017 | 0.025 | 0.027 | 0.022 | 0.020 | 0.025 | 0.028 | 0.026 | 0.024 |
| *Starkeya* | 0.016 | 0.017 | 0.022 | 0.018 | 0.019 | 0.022 | 0.029 | 0.030 | 0.033 | 0.023 |
| *Brevundimonas* | 0.025 | 0.020 | 0.023 | 0.013 | 0.020 | 0.015 | 0.022 | 0.031 | 0.030 | 0.022 |
| *Candidatus Accumulibacter* | 0.025 | 0.020 | 0.019 | 0.019 | 0.018 | 0.018 | 0.026 | 0.028 | 0.024 | 0.022 |
| *Stenotrophomonas* | 0.019 | 0.016 | 0.018 | 0.021 | 0.026 | 0.027 | 0.018 | 0.023 | 0.020 | 0.021 |
| *Gordonia* | 0.012 | 0.017 | 0.011 | 0.027 | 0.031 | 0.036 | 0.020 | 0.017 | 0.018 | 0.021 |
| *Nitrobacter* | 0.013 | 0.014 | 0.018 | 0.056 | 0.017 | 0.015 | 0.019 | 0.017 | 0.015 | 0.020 |
| *Achromobacter* | 0.024 | 0.018 | 0.020 | 0.014 | 0.017 | 0.021 | 0.017 | 0.026 | 0.025 | 0.020 |
| *Thauera* | 0.017 | 0.017 | 0.015 | 0.019 | 0.023 | 0.023 | 0.018 | 0.024 | 0.023 | 0.020 |
| *Kitasatospora* | 0.011 | 0.006 | 0.007 | 0.025 | 0.023 | 0.034 | 0.020 | 0.023 | 0.027 | 0.020 |
| *Nocardioides* | 0.009 | 0.013 | 0.010 | 0.032 | 0.028 | 0.029 | 0.016 | 0.018 | 0.019 | 0.020 |
| *Aromatoleum* | 0.016 | 0.021 | 0.027 | 0.022 | 0.020 | 0.024 | 0.016 | 0.015 | 0.014 | 0.019 |
| *Thermus* | 0.012 | 0.017 | 0.021 | 0.023 | 0.025 | 0.028 | 0.018 | 0.014 | 0.016 | 0.019 |
| *Thermaerobacter* | 0.011 | 0.012 | 0.008 | 0.029 | 0.043 | 0.036 | 0.010 | 0.012 | 0.011 | 0.019 |
| *Opitutus* | 0.016 | 0.013 | 0.016 | 0.020 | 0.020 | 0.018 | 0.017 | 0.027 | 0.025 | 0.019 |
| *Candidatus Chloracidobacterium* | 0.017 | 0.022 | 0.023 | 0.019 | 0.024 | 0.021 | 0.012 | 0.016 | 0.017 | 0.019 |
| *Sordaria* | 0.023 | 0.015 | 0.018 | 0.014 | 0.018 | 0.013 | 0.020 | 0.025 | 0.021 | 0.018 |
| *Actinosynnema* | 0.012 | 0.019 | 0.018 | 0.021 | 0.024 | 0.020 | 0.014 | 0.017 | 0.016 | 0.018 |
| *Tetrapisispora* | 0.015 | 0.018 | 0.019 | 0.018 | 0.017 | 0.021 | 0.016 | 0.015 | 0.017 | 0.017 |
| *Isoptericola* | 0.008 | 0.008 | 0.006 | 0.027 | 0.031 | 0.027 | 0.016 | 0.017 | 0.015 | 0.017 |
| *Tsukamurella* | 0.014 | 0.018 | 0.020 | 0.014 | 0.015 | 0.017 | 0.022 | 0.020 | 0.015 | 0.017 |
| *Rhodothermus* | 0.008 | 0.005 | 0.007 | 0.019 | 0.020 | 0.018 | 0.019 | 0.023 | 0.030 | 0.017 |
| *Leptothrix* | 0.007 | 0.010 | 0.012 | 0.020 | 0.023 | 0.020 | 0.016 | 0.019 | 0.021 | 0.016 |
| *Rhodobacter* | 0.014 | 0.013 | 0.016 | 0.014 | 0.020 | 0.017 | 0.013 | 0.020 | 0.021 | 0.016 |
| *Acidobacterium* | 0.012 | 0.016 | 0.019 | 0.011 | 0.017 | 0.020 | 0.015 | 0.015 | 0.021 | 0.016 |
| *Acidiphilium* | 0.014 | 0.016 | 0.016 | 0.015 | 0.014 | 0.019 | 0.012 | 0.019 | 0.019 | 0.016 |
| *Paenibacillus* | 0.015 | 0.007 | 0.013 | 0.015 | 0.017 | 0.018 | 0.018 | 0.017 | 0.024 | 0.016 |
| *Methylibium* | 0.018 | 0.016 | 0.014 | 0.013 | 0.015 | 0.019 | 0.016 | 0.016 | 0.017 | 0.016 |
| *Aeromonas* | 0.015 | 0.010 | 0.014 | 0.016 | 0.021 | 0.024 | 0.013 | 0.014 | 0.014 | 0.016 |
| *Hyphomicrobium* | 0.013 | 0.017 | 0.020 | 0.020 | 0.016 | 0.018 | 0.009 | 0.014 | 0.014 | 0.016 |
| *Janthinobacterium* | 0.016 | 0.019 | 0.012 | 0.017 | 0.015 | 0.022 | 0.010 | 0.013 | 0.016 | 0.016 |
| *Kutzneria* | 0.010 | 0.015 | 0.011 | 0.018 | 0.025 | 0.021 | 0.013 | 0.015 | 0.011 | 0.015 |
| *Acidimicrobium* | 0.006 | 0.009 | 0.006 | 0.026 | 0.029 | 0.030 | 0.009 | 0.010 | 0.010 | 0.015 |
| *Geodermatophilus* | 0.011 | 0.011 | 0.008 | 0.028 | 0.021 | 0.026 | 0.011 | 0.011 | 0.009 | 0.015 |
| *Verrucosispora* | 0.007 | 0.006 | 0.008 | 0.024 | 0.025 | 0.026 | 0.012 | 0.008 | 0.012 | 0.014 |
| *Corallococcus* | 0.010 | 0.005 | 0.008 | 0.008 | 0.007 | 0.014 | 0.017 | 0.027 | 0.034 | 0.014 |
| *Alicyclobacillus* | 0.011 | 0.016 | 0.015 | 0.013 | 0.019 | 0.017 | 0.010 | 0.016 | 0.011 | 0.014 |
| *Rhodospirillum* | 0.008 | 0.010 | 0.011 | 0.021 | 0.021 | 0.020 | 0.013 | 0.013 | 0.009 | 0.014 |
| *Corynebacterium* | 0.005 | 0.008 | 0.006 | 0.023 | 0.024 | 0.032 | 0.007 | 0.011 | 0.007 | 0.014 |
| *Thioalkalivibrio* | 0.010 | 0.014 | 0.012 | 0.014 | 0.015 | 0.015 | 0.013 | 0.015 | 0.016 | 0.014 |
| *Thiomonas* | 0.012 | 0.014 | 0.014 | 0.010 | 0.013 | 0.020 | 0.012 | 0.014 | 0.014 | 0.014 |
| *Gloeobacter* | 0.013 | 0.018 | 0.016 | 0.012 | 0.021 | 0.011 | 0.010 | 0.014 | 0.009 | 0.014 |
| *Thermomonospora* | 0.006 | 0.006 | 0.005 | 0.027 | 0.019 | 0.037 | 0.009 | 0.008 | 0.008 | 0.014 |
| *Phycisphaera* | 0.008 | 0.008 | 0.010 | 0.009 | 0.008 | 0.014 | 0.016 | 0.021 | 0.028 | 0.014 |
| *Acidithiobacillus* | 0.009 | 0.012 | 0.016 | 0.014 | 0.011 | 0.014 | 0.017 | 0.013 | 0.015 | 0.013 |
| *Magnetospirillum* | 0.011 | 0.009 | 0.014 | 0.009 | 0.014 | 0.014 | 0.015 | 0.016 | 0.020 | 0.013 |
| *Ramlibacter* | 0.010 | 0.011 | 0.014 | 0.012 | 0.013 | 0.013 | 0.014 | 0.015 | 0.017 | 0.013 |
| *Salinarchaeum* | 0.009 | 0.016 | 0.013 | 0.011 | 0.013 | 0.013 | 0.013 | 0.014 | 0.015 | 0.013 |
| *Candidatus Phytoplasma* | 0.000 | 0.102 | 0.012 | 0.000 | 0.000 | 0.000 | 0.000 | 0.000 | 0.000 | 0.013 |
| *Agrobacterium* | 0.012 | 0.009 | 0.014 | 0.010 | 0.008 | 0.011 | 0.012 | 0.015 | 0.022 | 0.013 |
| *Intrasporangium* | 0.007 | 0.006 | 0.004 | 0.015 | 0.019 | 0.016 | 0.013 | 0.012 | 0.017 | 0.012 |
| *Desulfovibrio* | 0.011 | 0.008 | 0.008 | 0.014 | 0.015 | 0.010 | 0.011 | 0.015 | 0.015 | 0.012 |
| *Saccharopolyspora* | 0.007 | 0.007 | 0.007 | 0.018 | 0.019 | 0.020 | 0.008 | 0.008 | 0.011 | 0.012 |
| *Thermobacillus* | 0.007 | 0.007 | 0.008 | 0.014 | 0.024 | 0.021 | 0.010 | 0.008 | 0.007 | 0.012 |
| *Ruegeria* | 0.012 | 0.011 | 0.007 | 0.009 | 0.016 | 0.013 | 0.008 | 0.010 | 0.015 | 0.011 |
| *Xanthobacter* | 0.009 | 0.007 | 0.011 | 0.022 | 0.011 | 0.017 | 0.009 | 0.009 | 0.008 | 0.011 |
| *Oligotropha* | 0.007 | 0.009 | 0.009 | 0.008 | 0.010 | 0.013 | 0.010 | 0.016 | 0.018 | 0.011 |
| *Melioribacter* | 0.010 | 0.013 | 0.015 | 0.008 | 0.011 | 0.011 | 0.010 | 0.008 | 0.010 | 0.011 |
| *Pseudoxanthomonas* | 0.010 | 0.005 | 0.005 | 0.007 | 0.006 | 0.007 | 0.011 | 0.020 | 0.024 | 0.011 |
| *Stackebrandtia* | 0.006 | 0.002 | 0.003 | 0.004 | 0.004 | 0.005 | 0.017 | 0.023 | 0.027 | 0.010 |
| *Saccharomonospora* | 0.008 | 0.015 | 0.010 | 0.010 | 0.007 | 0.008 | 0.011 | 0.013 | 0.010 | 0.010 |
| *Salinispora* | 0.005 | 0.007 | 0.005 | 0.017 | 0.019 | 0.018 | 0.008 | 0.006 | 0.005 | 0.010 |
| *Pseudogulbenkiania* | 0.007 | 0.010 | 0.006 | 0.011 | 0.012 | 0.016 | 0.006 | 0.010 | 0.012 | 0.010 |
| *Singulisphaera* | 0.004 | 0.009 | 0.010 | 0.011 | 0.014 | 0.015 | 0.010 | 0.008 | 0.008 | 0.010 |
| *Saccharothrix* | 0.005 | 0.007 | 0.006 | 0.014 | 0.016 | 0.021 | 0.006 | 0.008 | 0.007 | 0.010 |
| *Meyerozyma* | 0.000 | 0.000 | 0.000 | 0.024 | 0.025 | 0.040 | 0.000 | 0.000 | 0.000 | 0.010 |
| *Leptospirillum* | 0.008 | 0.006 | 0.014 | 0.006 | 0.010 | 0.009 | 0.016 | 0.010 | 0.008 | 0.010 |
| *Pelobacter* | 0.010 | 0.012 | 0.011 | 0.007 | 0.011 | 0.012 | 0.008 | 0.009 | 0.008 | 0.010 |
| *Marinithermus* | 0.011 | 0.011 | 0.007 | 0.008 | 0.016 | 0.010 | 0.007 | 0.009 | 0.007 | 0.010 |
| *Cytophaga* | 0.009 | 0.014 | 0.007 | 0.007 | 0.009 | 0.013 | 0.005 | 0.011 | 0.011 | 0.009 |
| *Natrinema* | 0.006 | 0.013 | 0.010 | 0.007 | 0.013 | 0.014 | 0.006 | 0.009 | 0.007 | 0.009 |
| *Gluconacetobacter* | 0.008 | 0.010 | 0.008 | 0.009 | 0.008 | 0.008 | 0.011 | 0.009 | 0.011 | 0.009 |
| *Thermobispora* | 0.009 | 0.004 | 0.004 | 0.010 | 0.009 | 0.007 | 0.010 | 0.016 | 0.014 | 0.009 |
| *Methylocystis* | 0.006 | 0.005 | 0.006 | 0.008 | 0.005 | 0.004 | 0.011 | 0.016 | 0.019 | 0.009 |
| *Micrococcus* | 0.005 | 0.004 | 0.006 | 0.013 | 0.014 | 0.015 | 0.007 | 0.008 | 0.010 | 0.009 |
| *Herbaspirillum* | 0.009 | 0.008 | 0.008 | 0.008 | 0.010 | 0.015 | 0.006 | 0.010 | 0.008 | 0.009 |
| *Neorhizobium* | 0.007 | 0.007 | 0.008 | 0.006 | 0.012 | 0.017 | 0.007 | 0.007 | 0.010 | 0.009 |
| *Azospira* | 0.010 | 0.006 | 0.009 | 0.008 | 0.008 | 0.008 | 0.007 | 0.010 | 0.012 | 0.009 |
| *Chlorobaculum* | 0.005 | 0.004 | 0.004 | 0.018 | 0.015 | 0.016 | 0.003 | 0.006 | 0.005 | 0.009 |
| *Nitrosospira* | 0.007 | 0.009 | 0.008 | 0.007 | 0.012 | 0.012 | 0.007 | 0.006 | 0.009 | 0.008 |
| *Arthrobacter* | 0.005 | 0.005 | 0.003 | 0.011 | 0.011 | 0.013 | 0.007 | 0.010 | 0.010 | 0.008 |
| *Pelodictyon* | 0.007 | 0.010 | 0.011 | 0.008 | 0.011 | 0.009 | 0.007 | 0.006 | 0.005 | 0.008 |
| *Candidatus Methylomirabilis* | 0.007 | 0.010 | 0.008 | 0.009 | 0.008 | 0.009 | 0.008 | 0.008 | 0.007 | 0.008 |
| *Rubrivivax* | 0.006 | 0.003 | 0.005 | 0.008 | 0.005 | 0.009 | 0.009 | 0.012 | 0.015 | 0.008 |
| *Propionibacterium* | 0.004 | 0.003 | 0.004 | 0.016 | 0.015 | 0.013 | 0.003 | 0.006 | 0.005 | 0.008 |
| *Wallemia* | 0.000 | 0.000 | 0.000 | 0.028 | 0.028 | 0.015 | 0.000 | 0.000 | 0.000 | 0.008 |
| *Spirochaeta* | 0.006 | 0.009 | 0.011 | 0.007 | 0.006 | 0.007 | 0.005 | 0.009 | 0.009 | 0.008 |
| *Paracoccus* | 0.010 | 0.005 | 0.007 | 0.010 | 0.011 | 0.014 | 0.003 | 0.004 | 0.003 | 0.008 |
| *Fibrella* | 0.012 | 0.007 | 0.008 | 0.007 | 0.007 | 0.005 | 0.006 | 0.010 | 0.007 | 0.008 |
| *Thiobacillus* | 0.005 | 0.004 | 0.006 | 0.008 | 0.009 | 0.008 | 0.009 | 0.007 | 0.010 | 0.007 |
| *Thermomicrobium* | 0.007 | 0.008 | 0.005 | 0.008 | 0.009 | 0.007 | 0.006 | 0.009 | 0.009 | 0.007 |
| *Xylanimonas* | 0.003 | 0.004 | 0.003 | 0.009 | 0.013 | 0.018 | 0.005 | 0.007 | 0.006 | 0.007 |
| *Natronococcus* | 0.005 | 0.003 | 0.004 | 0.005 | 0.004 | 0.002 | 0.011 | 0.015 | 0.018 | 0.007 |
| *Chromobacterium* | 0.005 | 0.002 | 0.003 | 0.005 | 0.004 | 0.005 | 0.010 | 0.015 | 0.015 | 0.007 |
| *Alistipes* | 0.005 | 0.010 | 0.010 | 0.006 | 0.008 | 0.010 | 0.004 | 0.006 | 0.006 | 0.007 |
| *Desulfurispirillum* | 0.007 | 0.008 | 0.009 | 0.008 | 0.009 | 0.008 | 0.004 | 0.006 | 0.005 | 0.007 |
| *Gaeumannomyces* | 0.004 | 0.001 | 0.003 | 0.001 | 0.001 | 0.001 | 0.013 | 0.019 | 0.021 | 0.007 |
| *Cronobacter* | 0.008 | 0.007 | 0.006 | 0.008 | 0.008 | 0.008 | 0.005 | 0.006 | 0.006 | 0.007 |
| *Sulfuritalea* | 0.005 | 0.004 | 0.005 | 0.011 | 0.012 | 0.011 | 0.004 | 0.005 | 0.006 | 0.007 |
| *Acidovorax* | 0.005 | 0.007 | 0.005 | 0.006 | 0.008 | 0.007 | 0.007 | 0.009 | 0.007 | 0.007 |
| *Polaromonas* | 0.005 | 0.005 | 0.005 | 0.012 | 0.005 | 0.008 | 0.007 | 0.006 | 0.006 | 0.007 |
| *Leptolyngbya* | 0.006 | 0.009 | 0.014 | 0.004 | 0.007 | 0.008 | 0.005 | 0.003 | 0.003 | 0.007 |
| *Caldilinea* | 0.006 | 0.006 | 0.004 | 0.013 | 0.007 | 0.007 | 0.004 | 0.005 | 0.006 | 0.007 |
| *Parvibaculum* | 0.006 | 0.004 | 0.009 | 0.004 | 0.007 | 0.009 | 0.008 | 0.007 | 0.005 | 0.006 |
| *Micavibrio* | 0.007 | 0.005 | 0.007 | 0.006 | 0.005 | 0.004 | 0.008 | 0.009 | 0.007 | 0.006 |
| *Sideroxydans* | 0.005 | 0.002 | 0.003 | 0.012 | 0.007 | 0.010 | 0.004 | 0.005 | 0.005 | 0.006 |
| *Methanocella* | 0.004 | 0.010 | 0.006 | 0.005 | 0.004 | 0.004 | 0.007 | 0.006 | 0.007 | 0.006 |
| *Escherichia* | 0.006 | 0.008 | 0.006 | 0.003 | 0.004 | 0.003 | 0.005 | 0.008 | 0.008 | 0.006 |
| *Nostoc* | 0.004 | 0.007 | 0.012 | 0.006 | 0.003 | 0.006 | 0.003 | 0.005 | 0.005 | 0.006 |
| *Gordonibacter* | 0.004 | 0.001 | 0.004 | 0.002 | 0.002 | 0.003 | 0.007 | 0.010 | 0.014 | 0.005 |
| *Thiocystis* | 0.003 | 0.005 | 0.004 | 0.003 | 0.006 | 0.005 | 0.007 | 0.007 | 0.008 | 0.005 |
| *Castellaniella* | 0.004 | 0.005 | 0.004 | 0.003 | 0.007 | 0.008 | 0.007 | 0.005 | 0.004 | 0.005 |
| *Methylophaga* | 0.008 | 0.005 | 0.005 | 0.002 | 0.004 | 0.004 | 0.005 | 0.006 | 0.005 | 0.005 |
| *Niastella* | 0.004 | 0.007 | 0.001 | 0.007 | 0.007 | 0.008 | 0.003 | 0.005 | 0.004 | 0.005 |
| *Halorubrum* | 0.004 | 0.004 | 0.006 | 0.003 | 0.005 | 0.007 | 0.004 | 0.005 | 0.007 | 0.005 |
| *Delftia* | 0.003 | 0.002 | 0.002 | 0.002 | 0.001 | 0.003 | 0.005 | 0.012 | 0.014 | 0.005 |
| *Ensifer* | 0.003 | 0.003 | 0.006 | 0.005 | 0.005 | 0.005 | 0.006 | 0.005 | 0.005 | 0.005 |
| *Acaryochloris* | 0.005 | 0.005 | 0.008 | 0.003 | 0.004 | 0.004 | 0.006 | 0.004 | 0.004 | 0.005 |
| *Sulfuricella* | 0.004 | 0.005 | 0.005 | 0.005 | 0.004 | 0.008 | 0.005 | 0.005 | 0.003 | 0.005 |
| *Candidatus Saccharimonas* | 0.001 | 0.001 | 0.001 | 0.011 | 0.013 | 0.014 | 0.001 | 0.000 | 0.000 | 0.005 |
| *Marichromatium* | 0.001 | 0.000 | 0.003 | 0.001 | 0.001 | 0.001 | 0.009 | 0.011 | 0.014 | 0.005 |
| *Bifidobacterium* | 0.003 | 0.002 | 0.003 | 0.003 | 0.004 | 0.007 | 0.003 | 0.006 | 0.007 | 0.004 |
| *Microbacterium* | 0.001 | 0.003 | 0.001 | 0.007 | 0.005 | 0.009 | 0.004 | 0.004 | 0.005 | 0.004 |
| *Haloferax* | 0.002 | 0.001 | 0.002 | 0.001 | 0.001 | 0.001 | 0.008 | 0.009 | 0.012 | 0.004 |
| *Candida* | 0.004 | 0.005 | 0.003 | 0.005 | 0.004 | 0.007 | 0.003 | 0.004 | 0.004 | 0.004 |
| *Oceanithermus* | 0.003 | 0.002 | 0.004 | 0.004 | 0.003 | 0.004 | 0.003 | 0.006 | 0.009 | 0.004 |
| *Desulfarculus* | 0.004 | 0.003 | 0.006 | 0.005 | 0.004 | 0.006 | 0.003 | 0.003 | 0.003 | 0.004 |
| *Roseibacterium* | 0.004 | 0.004 | 0.003 | 0.003 | 0.003 | 0.003 | 0.004 | 0.007 | 0.005 | 0.004 |
| *Synechococcus* | 0.001 | 0.001 | 0.002 | 0.007 | 0.009 | 0.010 | 0.002 | 0.002 | 0.002 | 0.004 |
| *Dermacoccus* | 0.002 | 0.002 | 0.001 | 0.005 | 0.005 | 0.004 | 0.005 | 0.005 | 0.008 | 0.004 |
| *Verminephrobacter* | 0.003 | 0.002 | 0.005 | 0.004 | 0.005 | 0.003 | 0.005 | 0.004 | 0.005 | 0.004 |
| *Methanoculleus* | 0.002 | 0.001 | 0.001 | 0.004 | 0.002 | 0.003 | 0.005 | 0.009 | 0.009 | 0.004 |
| *Pyrenophora* | 0.002 | 0.004 | 0.002 | 0.004 | 0.005 | 0.006 | 0.002 | 0.005 | 0.004 | 0.004 |
| *Thermobifida* | 0.003 | 0.002 | 0.005 | 0.004 | 0.004 | 0.004 | 0.004 | 0.003 | 0.006 | 0.004 |
| *Pandoraea* | 0.006 | 0.003 | 0.004 | 0.007 | 0.004 | 0.003 | 0.002 | 0.002 | 0.002 | 0.004 |
| *Salinibacter* | 0.004 | 0.004 | 0.003 | 0.004 | 0.005 | 0.006 | 0.004 | 0.002 | 0.001 | 0.004 |
| *Microlunatus* | 0.002 | 0.003 | 0.002 | 0.005 | 0.007 | 0.009 | 0.001 | 0.003 | 0.002 | 0.004 |
| *Pluralibacter* | 0.003 | 0.002 | 0.004 | 0.003 | 0.004 | 0.002 | 0.005 | 0.005 | 0.004 | 0.004 |
| *Meiothermus* | 0.001 | 0.002 | 0.003 | 0.003 | 0.005 | 0.004 | 0.004 | 0.003 | 0.007 | 0.004 |
| *Leptosphaeria* | 0.000 | 0.000 | 0.000 | 0.009 | 0.011 | 0.012 | 0.000 | 0.000 | 0.000 | 0.004 |
| *Epichloe* | 0.002 | 0.001 | 0.001 | 0.001 | 0.000 | 0.001 | 0.004 | 0.010 | 0.011 | 0.003 |
| *Candidatus Kuenenia* | 0.003 | 0.002 | 0.005 | 0.002 | 0.003 | 0.004 | 0.005 | 0.004 | 0.004 | 0.003 |
| *Trueperella* | 0.003 | 0.004 | 0.003 | 0.003 | 0.006 | 0.003 | 0.002 | 0.003 | 0.004 | 0.003 |
| *Prevotella* | 0.004 | 0.004 | 0.004 | 0.002 | 0.003 | 0.004 | 0.002 | 0.004 | 0.003 | 0.003 |
| *Haloarcula* | 0.005 | 0.003 | 0.005 | 0.002 | 0.004 | 0.002 | 0.004 | 0.003 | 0.003 | 0.003 |
| *Kineococcus* | 0.002 | 0.004 | 0.002 | 0.005 | 0.008 | 0.003 | 0.002 | 0.002 | 0.002 | 0.003 |
| *Cladophialophora* | 0.000 | 0.002 | 0.001 | 0.006 | 0.009 | 0.005 | 0.001 | 0.002 | 0.002 | 0.003 |
| *Eggerthella* | 0.002 | 0.003 | 0.004 | 0.002 | 0.004 | 0.003 | 0.003 | 0.004 | 0.003 | 0.003 |
| *Methylococcus* | 0.004 | 0.001 | 0.002 | 0.005 | 0.004 | 0.005 | 0.003 | 0.003 | 0.002 | 0.003 |
| *Komagataeibacter* | 0.002 | 0.003 | 0.003 | 0.001 | 0.003 | 0.003 | 0.005 | 0.005 | 0.003 | 0.003 |
| *Desulfobulbus* | 0.003 | 0.000 | 0.001 | 0.000 | 0.000 | 0.001 | 0.004 | 0.010 | 0.010 | 0.003 |
| *Chloroflexus* | 0.004 | 0.003 | 0.004 | 0.001 | 0.004 | 0.001 | 0.003 | 0.004 | 0.004 | 0.003 |
| *Azorhizobium* | 0.001 | 0.001 | 0.001 | 0.009 | 0.004 | 0.006 | 0.001 | 0.002 | 0.002 | 0.003 |
| *Halomicrobium* | 0.002 | 0.002 | 0.002 | 0.003 | 0.005 | 0.004 | 0.003 | 0.002 | 0.003 | 0.003 |
| *Alicycliphilus* | 0.002 | 0.005 | 0.002 | 0.002 | 0.003 | 0.003 | 0.002 | 0.004 | 0.003 | 0.003 |
| *Cordyceps* | 0.003 | 0.001 | 0.001 | 0.004 | 0.006 | 0.007 | 0.001 | 0.002 | 0.002 | 0.003 |
| *Enterobacter* | 0.003 | 0.002 | 0.006 | 0.001 | 0.005 | 0.001 | 0.002 | 0.003 | 0.002 | 0.003 |
| *Chthonomonas* | 0.003 | 0.004 | 0.003 | 0.001 | 0.003 | 0.005 | 0.002 | 0.002 | 0.002 | 0.003 |
| *Sanguibacter* | 0.001 | 0.002 | 0.005 | 0.002 | 0.004 | 0.003 | 0.004 | 0.002 | 0.003 | 0.003 |
| *Chelativorans* | 0.001 | 0.001 | 0.002 | 0.004 | 0.006 | 0.005 | 0.002 | 0.002 | 0.002 | 0.003 |
| *Aspergillus* | 0.003 | 0.002 | 0.003 | 0.002 | 0.002 | 0.002 | 0.003 | 0.005 | 0.004 | 0.003 |
| *Halorhabdus* | 0.003 | 0.001 | 0.002 | 0.003 | 0.002 | 0.002 | 0.004 | 0.003 | 0.006 | 0.003 |
| *Rhodomicrobium* | 0.002 | 0.002 | 0.004 | 0.004 | 0.002 | 0.004 | 0.003 | 0.002 | 0.003 | 0.003 |
| *Syntrophobacter* | 0.002 | 0.002 | 0.002 | 0.002 | 0.003 | 0.003 | 0.003 | 0.003 | 0.003 | 0.003 |
| *Methylocella* | 0.002 | 0.002 | 0.001 | 0.008 | 0.002 | 0.002 | 0.002 | 0.002 | 0.002 | 0.003 |
| *Natrialba* | 0.002 | 0.002 | 0.002 | 0.005 | 0.003 | 0.003 | 0.001 | 0.001 | 0.003 | 0.002 |
| *Geobacillus* | 0.001 | 0.002 | 0.003 | 0.002 | 0.003 | 0.002 | 0.003 | 0.003 | 0.002 | 0.002 |
| *Runella* | 0.001 | 0.003 | 0.003 | 0.002 | 0.002 | 0.002 | 0.002 | 0.003 | 0.004 | 0.002 |
| *Nitrospira* | 0.001 | 0.002 | 0.001 | 0.001 | 0.001 | 0.003 | 0.002 | 0.003 | 0.005 | 0.002 |
| *Desulfococcus* | 0.002 | 0.003 | 0.002 | 0.003 | 0.001 | 0.005 | 0.002 | 0.001 | 0.001 | 0.002 |
| *Turneriella* | 0.003 | 0.002 | 0.002 | 0.001 | 0.001 | 0.002 | 0.002 | 0.004 | 0.004 | 0.002 |
| *Calonectria* | 0.000 | 0.000 | 0.000 | 0.005 | 0.005 | 0.010 | 0.000 | 0.000 | 0.000 | 0.002 |
| *Allochromatium* | 0.002 | 0.001 | 0.002 | 0.003 | 0.003 | 0.004 | 0.001 | 0.002 | 0.001 | 0.002 |
| *Endocarpon* | 0.000 | 0.000 | 0.000 | 0.019 | 0.000 | 0.000 | 0.000 | 0.000 | 0.000 | 0.002 |
| *Beutenbergia* | 0.000 | 0.001 | 0.001 | 0.002 | 0.005 | 0.002 | 0.001 | 0.003 | 0.002 | 0.002 |
| *Acidothermus* | 0.001 | 0.002 | 0.000 | 0.003 | 0.005 | 0.003 | 0.002 | 0.002 | 0.001 | 0.002 |
| *Verticillium* | 0.002 | 0.000 | 0.000 | 0.003 | 0.001 | 0.007 | 0.002 | 0.002 | 0.000 | 0.002 |
| *Coriobacterium* | 0.002 | 0.003 | 0.003 | 0.000 | 0.002 | 0.001 | 0.001 | 0.002 | 0.003 | 0.002 |
| *Slackia* | 0.001 | 0.002 | 0.002 | 0.001 | 0.002 | 0.002 | 0.002 | 0.003 | 0.003 | 0.002 |
| *Haloterrigena* | 0.000 | 0.001 | 0.001 | 0.004 | 0.003 | 0.003 | 0.000 | 0.002 | 0.002 | 0.002 |
| *Setosphaeria* | 0.000 | 0.001 | 0.001 | 0.000 | 0.000 | 0.000 | 0.002 | 0.006 | 0.005 | 0.002 |
| *Sclerotinia* | 0.000 | 0.002 | 0.000 | 0.001 | 0.002 | 0.001 | 0.002 | 0.003 | 0.003 | 0.002 |
| *Shewanella* | 0.001 | 0.003 | 0.002 | 0.002 | 0.002 | 0.002 | 0.001 | 0.001 | 0.001 | 0.002 |
| *Archaeoglobus* | 0.002 | 0.001 | 0.001 | 0.003 | 0.002 | 0.002 | 0.002 | 0.002 | 0.001 | 0.002 |
| *Scheffersomyces* | 0.000 | 0.001 | 0.002 | 0.002 | 0.003 | 0.001 | 0.003 | 0.002 | 0.001 | 0.002 |
| *Trametes* | 0.001 | 0.000 | 0.001 | 0.000 | 0.001 | 0.000 | 0.002 | 0.005 | 0.005 | 0.002 |
| *Olsenella* | 0.001 | 0.000 | 0.002 | 0.003 | 0.002 | 0.002 | 0.002 | 0.002 | 0.002 | 0.002 |
| *Kocuria* | 0.000 | 0.001 | 0.001 | 0.002 | 0.001 | 0.003 | 0.002 | 0.002 | 0.002 | 0.002 |
| *Roseobacter* | 0.002 | 0.002 | 0.002 | 0.001 | 0.000 | 0.001 | 0.002 | 0.002 | 0.003 | 0.002 |
| *Klebsiella* | 0.004 | 0.002 | 0.002 | 0.001 | 0.002 | 0.001 | 0.001 | 0.001 | 0.001 | 0.002 |
| *Eutypa* | 0.001 | 0.002 | 0.002 | 0.001 | 0.001 | 0.001 | 0.001 | 0.002 | 0.003 | 0.002 |
| *Porphyromonas* | 0.000 | 0.001 | 0.001 | 0.002 | 0.002 | 0.002 | 0.002 | 0.002 | 0.001 | 0.002 |
| *Bacteroides* | 0.001 | 0.001 | 0.001 | 0.001 | 0.002 | 0.004 | 0.001 | 0.002 | 0.001 | 0.002 |
| *Brucella* | 0.002 | 0.001 | 0.002 | 0.001 | 0.000 | 0.004 | 0.001 | 0.002 | 0.001 | 0.002 |
| *Rhodoferax* | 0.001 | 0.001 | 0.001 | 0.002 | 0.002 | 0.002 | 0.001 | 0.002 | 0.001 | 0.002 |
| *Trichoderma* | 0.002 | 0.001 | 0.000 | 0.004 | 0.003 | 0.003 | 0.000 | 0.000 | 0.000 | 0.001 |
| *Nonomuraea* | 0.001 | 0.000 | 0.001 | 0.000 | 0.000 | 0.000 | 0.004 | 0.003 | 0.004 | 0.001 |
| *Thermobaculum* | 0.001 | 0.001 | 0.000 | 0.002 | 0.004 | 0.002 | 0.001 | 0.001 | 0.001 | 0.001 |
| *Amycolicicoccus* | 0.001 | 0.001 | 0.000 | 0.004 | 0.002 | 0.002 | 0.002 | 0.001 | 0.001 | 0.001 |
| *Hyphomonas* | 0.001 | 0.000 | 0.001 | 0.002 | 0.002 | 0.002 | 0.001 | 0.002 | 0.002 | 0.001 |
| *Chaetomium* | 0.001 | 0.000 | 0.000 | 0.000 | 0.000 | 0.000 | 0.003 | 0.004 | 0.004 | 0.001 |
| *Dactylosporangium* | 0.001 | 0.001 | 0.001 | 0.001 | 0.002 | 0.002 | 0.001 | 0.002 | 0.002 | 0.001 |
| *Tistrella* | 0.003 | 0.001 | 0.001 | 0.001 | 0.001 | 0.001 | 0.001 | 0.001 | 0.002 | 0.001 |
| *Simiduia* | 0.000 | 0.001 | 0.000 | 0.001 | 0.001 | 0.002 | 0.002 | 0.002 | 0.002 | 0.001 |
| *Frateuria* | 0.001 | 0.000 | 0.000 | 0.001 | 0.002 | 0.003 | 0.001 | 0.002 | 0.001 | 0.001 |
| *Pelagibacterium* | 0.002 | 0.002 | 0.000 | 0.002 | 0.001 | 0.001 | 0.001 | 0.001 | 0.001 | 0.001 |
| *Selenomonas* | 0.001 | 0.001 | 0.001 | 0.001 | 0.001 | 0.002 | 0.002 | 0.001 | 0.001 | 0.001 |
| *Odoribacter* | 0.001 | 0.001 | 0.001 | 0.001 | 0.000 | 0.001 | 0.003 | 0.002 | 0.000 | 0.001 |
| *Kytococcus* | 0.002 | 0.001 | 0.001 | 0.002 | 0.001 | 0.001 | 0.001 | 0.001 | 0.001 | 0.001 |
| *Octadecabacter* | 0.003 | 0.000 | 0.003 | 0.001 | 0.001 | 0.001 | 0.001 | 0.000 | 0.000 | 0.001 |
| *Halovivax* | 0.001 | 0.000 | 0.001 | 0.000 | 0.000 | 0.000 | 0.001 | 0.003 | 0.004 | 0.001 |
| *Myceliophthora* | 0.001 | 0.000 | 0.000 | 0.000 | 0.000 | 0.001 | 0.004 | 0.002 | 0.002 | 0.001 |
| *Desulfohalobium* | 0.000 | 0.002 | 0.001 | 0.001 | 0.002 | 0.002 | 0.001 | 0.000 | 0.000 | 0.001 |
| *Parastagonospora* | 0.000 | 0.009 | 0.000 | 0.000 | 0.000 | 0.000 | 0.000 | 0.000 | 0.000 | 0.001 |
| *Thermodesulfatator* | 0.000 | 0.000 | 0.000 | 0.000 | 0.001 | 0.000 | 0.003 | 0.003 | 0.002 | 0.001 |
| *Ethanoligenens* | 0.002 | 0.001 | 0.001 | 0.003 | 0.001 | 0.002 | 0.000 | 0.000 | 0.000 | 0.001 |
| *Nitrososphaera* | 0.000 | 0.000 | 0.000 | 0.000 | 0.000 | 0.000 | 0.002 | 0.002 | 0.003 | 0.001 |
| *Granulibacter* | 0.000 | 0.000 | 0.000 | 0.009 | 0.000 | 0.000 | 0.000 | 0.000 | 0.000 | 0.001 |
| *Lysobacter* | 0.000 | 0.000 | 0.000 | 0.001 | 0.003 | 0.002 | 0.001 | 0.001 | 0.001 | 0.001 |
| *Chondromyces* | 0.001 | 0.001 | 0.001 | 0.001 | 0.002 | 0.001 | 0.000 | 0.001 | 0.000 | 0.001 |
| *Legionella* | 0.000 | 0.002 | 0.001 | 0.001 | 0.001 | 0.000 | 0.001 | 0.000 | 0.002 | 0.001 |
| *Hahella* | 0.001 | 0.001 | 0.001 | 0.001 | 0.001 | 0.000 | 0.000 | 0.001 | 0.002 | 0.001 |
| *Saccharophagus* | 0.001 | 0.000 | 0.001 | 0.000 | 0.000 | 0.000 | 0.002 | 0.002 | 0.002 | 0.001 |
| *Gluconobacter* | 0.001 | 0.002 | 0.001 | 0.000 | 0.001 | 0.002 | 0.000 | 0.000 | 0.000 | 0.001 |
| *Marinobacter* | 0.002 | 0.001 | 0.001 | 0.001 | 0.001 | 0.002 | 0.000 | 0.000 | 0.000 | 0.001 |
| *Faecalibacterium* | 0.000 | 0.000 | 0.000 | 0.002 | 0.004 | 0.000 | 0.000 | 0.000 | 0.000 | 0.001 |
| *Ochrobactrum* | 0.000 | 0.001 | 0.001 | 0.001 | 0.002 | 0.001 | 0.001 | 0.001 | 0.001 | 0.001 |
| *Thioflavicoccus* | 0.001 | 0.000 | 0.001 | 0.000 | 0.001 | 0.001 | 0.001 | 0.001 | 0.001 | 0.001 |
| *Comamonas* | 0.001 | 0.000 | 0.001 | 0.000 | 0.002 | 0.001 | 0.001 | 0.000 | 0.001 | 0.001 |
| *Lodderomyces* | 0.000 | 0.000 | 0.000 | 0.000 | 0.001 | 0.004 | 0.001 | 0.000 | 0.001 | 0.001 |
| *Asticcacaulis* | 0.000 | 0.000 | 0.001 | 0.001 | 0.000 | 0.001 | 0.001 | 0.001 | 0.001 | 0.001 |
| *Truepera* | 0.001 | 0.001 | 0.001 | 0.002 | 0.001 | 0.001 | 0.000 | 0.000 | 0.001 | 0.001 |
| *Serratia* | 0.003 | 0.000 | 0.003 | 0.000 | 0.000 | 0.000 | 0.000 | 0.001 | 0.000 | 0.001 |
| *Chitiniphilus* | 0.001 | 0.001 | 0.001 | 0.001 | 0.001 | 0.001 | 0.000 | 0.001 | 0.001 | 0.001 |
| *Dechloromonas* | 0.001 | 0.000 | 0.001 | 0.000 | 0.002 | 0.001 | 0.000 | 0.001 | 0.000 | 0.001 |
| *Ruminiclostridium* | 0.000 | 0.001 | 0.000 | 0.001 | 0.002 | 0.002 | 0.000 | 0.000 | 0.000 | 0.001 |
| *Candidatus Acetothermum* | 0.000 | 0.000 | 0.000 | 0.000 | 0.000 | 0.000 | 0.002 | 0.001 | 0.002 | 0.001 |
| *Alcanivorax* | 0.001 | 0.000 | 0.000 | 0.001 | 0.001 | 0.002 | 0.000 | 0.000 | 0.000 | 0.001 |
| *Thermosynechococcus* | 0.002 | 0.001 | 0.001 | 0.000 | 0.000 | 0.001 | 0.000 | 0.000 | 0.000 | 0.001 |
| *Niabella* | 0.000 | 0.000 | 0.000 | 0.003 | 0.001 | 0.001 | 0.000 | 0.000 | 0.000 | 0.001 |
| *Heliobacterium* | 0.001 | 0.000 | 0.000 | 0.001 | 0.001 | 0.001 | 0.001 | 0.001 | 0.000 | 0.001 |
| *Fimbriimonas* | 0.003 | 0.000 | 0.001 | 0.000 | 0.000 | 0.000 | 0.000 | 0.000 | 0.000 | 0.001 |
| *Halorhodospira* | 0.000 | 0.000 | 0.000 | 0.000 | 0.000 | 0.000 | 0.001 | 0.001 | 0.002 | 0.001 |
| *Rhodopirellula* | 0.000 | 0.000 | 0.000 | 0.001 | 0.002 | 0.001 | 0.000 | 0.000 | 0.000 | 0.001 |
| *Natronomonas* | 0.000 | 0.001 | 0.000 | 0.001 | 0.000 | 0.002 | 0.000 | 0.000 | 0.000 | 0.001 |
| *Bacillus* | 0.000 | 0.000 | 0.000 | 0.001 | 0.002 | 0.002 | 0.000 | 0.000 | 0.000 | 0.001 |
| *Leisingera* | 0.000 | 0.001 | 0.000 | 0.001 | 0.000 | 0.001 | 0.000 | 0.000 | 0.000 | 0.001 |
| *Herminiimonas* | 0.001 | 0.000 | 0.000 | 0.000 | 0.000 | 0.000 | 0.001 | 0.001 | 0.002 | 0.001 |
| *Neisseria* | 0.003 | 0.000 | 0.001 | 0.000 | 0.000 | 0.000 | 0.000 | 0.000 | 0.000 | 0.000 |
| *Glomus* | 0.001 | 0.000 | 0.001 | 0.001 | 0.000 | 0.000 | 0.000 | 0.000 | 0.000 | 0.000 |
| *Halobacterium* | 0.000 | 0.000 | 0.001 | 0.000 | 0.000 | 0.000 | 0.001 | 0.001 | 0.001 | 0.000 |
| *Dinoroseobacter* | 0.001 | 0.000 | 0.000 | 0.001 | 0.000 | 0.001 | 0.000 | 0.000 | 0.000 | 0.000 |
| *Halothiobacillus* | 0.000 | 0.000 | 0.001 | 0.001 | 0.002 | 0.000 | 0.000 | 0.000 | 0.000 | 0.000 |
| *Dyadobacter* | 0.000 | 0.000 | 0.000 | 0.000 | 0.000 | 0.001 | 0.000 | 0.000 | 0.002 | 0.000 |
| *Segniliparus* | 0.000 | 0.000 | 0.000 | 0.001 | 0.001 | 0.000 | 0.000 | 0.000 | 0.000 | 0.000 |
| *Idiomarina* | 0.000 | 0.000 | 0.000 | 0.001 | 0.000 | 0.000 | 0.000 | 0.000 | 0.000 | 0.000 |

**Supplementary Data S3.** The proportions (%) of compounds in soil extracts as determined using GC-MS with ribitol as internal standard. CK; Control; PN: *Paspalum natatum*; SG: *Stylosanthes guianensis*.

| Category | Compounds | PN1 | PN2 | PN3 | SG1 | SG2 | SG3 | CK1 | CK2 | CK3 |
| --- | --- | --- | --- | --- | --- | --- | --- | --- | --- | --- |
|  | Pentasiloxane | 0.255 | 0.000 | 0.000 | 0.740 | 0.000 | 0.000 | 1.048 | 0.350 | 0.929 |
|  | Urea | 0.226 | 0.000 | 0.000 | 0.000 | 0.000 | 0.000 | 0.000 | 0.000 | 0.000 |
|  | Hexasiloxane | 0.090 | 0.000 | 0.076 | 0.154 | 0.135 | 0.000 | 0.000 | 0.000 | 0.000 |
|  | Thiodiglycol | 0.000 | 0.153 | 0.000 | 0.000 | 0.000 | 0.597 | 0.000 | 0.000 | 0.000 |
|  | Nonadecanenitrile | 0.832 | 0.000 | 0.447 | 0.568 | 0.497 | 0.621 | 0.000 | 0.000 | 0.000 |
|  | Fluorene | 0.000 | 0.000 | 0.000 | 0.000 | 0.411 | 0.000 | 0.000 | 0.000 | 0.000 |
| Organic acid | Carbamic acid | 3.513 | 6.474 | 2.369 | 1.163 | 1.187 | 1.591 | 0.000 | 0.000 | 0.000 |
|  | Benzoic acid | 3.438 | 2.371 | 1.214 | 0.501 | 0.756 | 0.809 | 0.000 | 0.000 | 0.000 |
|  | Nonanoic acid | 0.000 | 0.000 | 0.000 | 0.077 | 0.083 | 0.094 | 0.000 | 0.000 | 0.000 |
|  | 1,4-Benzenedicarboxylic acid | 0.000 | 0.000 | 0.647 | 0.704 | 0.392 | 0.940 | 0.497 | 0.482 | 0.817 |
|  | Tetradecanoic acid | 1.316 | 1.164 | 1.324 | 1.066 | 1.405 | 1.007 | 0.929 | 1.452 | 0.726 |
|  | Hexadecanoic acid | 26.446 | 36.710 | 31.096 | 24.713 | 39.089 | 40.742 | 37.234 | 43.765 | 30.183 |
|  | 9,12-Octadecadienoic acid | 0.944 | 1.655 | 1.116 | 0.779 | 1.263 | 1.074 | 0.984 | 1.237 | 1.224 |
|  | 11-trans-Octadecenoic acid | 0.510 | 0.993 | 0.633 | 0.465 | 0.805 | 0.801 | 0.699 | 0.931 | 0.872 |
|  | Octadecanoic acid | 15.686 | 21.052 | 18.198 | 15.743 | 21.325 | 22.255 | 26.942 | 23.765 | 19.371 |
|  | Sulfurous acid | 0.576 | 0.473 | 0.527 | 0.000 | 0.670 | 0.458 | 0.000 | 0.000 | 0.000 |
| Amide | Acetamide | 0.784 | 3.398 | 0.000 | 4.062 | 0.000 | 0.000 | 1.013 | 0.000 | 3.841 |
|  | Hexadecanamide | 0.253 | 0.000 | 0.000 | 0.000 | 0.000 | 0.000 | 0.000 | 0.000 | 0.000 |
|  | 9-Octadecenamide | 0.415 | 1.373 | 0.520 | 0.396 | 0.592 | 2.399 | 0.781 | 1.530 | 0.000 |
|  | Oleamide | 0.506 | 1.950 | 0.512 | 2.150 | 0.000 | 0.000 | 0.000 | 0.000 | 1.521 |
|  | 13-Docosenamide | 2.356 | 0.000 | 0.664 | 0.000 | 0.000 | 0.000 | 0.000 | 0.000 | 0.000 |
| Amine | Ethylamine | 1.130 | 0.000 | 1.113 | 0.586 | 0.872 | 1.006 | 0.553 | 0.607 | 0.825 |
|  | Allyl(2,3-dihydro-1H-benzo[4,5]imidazo[1,2-a]cyclopenta[d]pyrimidin-11-yl)amine | 0.128 | 0.000 | 0.000 | 0.125 | 0.000 | 0.000 | 0.000 | 0.000 | 0.000 |
|  | Phenylenediamine | 0.000 | 0.000 | 0.000 | 0.550 | 0.000 | 0.000 | 0.847 | 0.000 | 0.000 |
| Carbohydrate | Glycerol | 6.219 | 6.165 | 5.948 | 6.304 | 6.496 | 6.265 | 6.631 | 7.109 | 3.638 |
|  | Xylulose | 0.000 | 0.337 | 0.000 | 0.321 | 0.000 | 0.000 | 0.000 | 0.000 | 0.000 |
|  | Fructose | 0.296 | 0.000 | 0.281 | 0.000 | 0.414 | 0.000 | 0.000 | 0.000 | 0.000 |
|  | Myo-Inositol | 0.807 | 0.992 | 0.752 | 0.581 | 1.093 | 0.839 | 0.000 | 0.000 | 0.000 |
|  | D-Mannitol | 0.000 | 0.000 | 0.000 | 0.000 | 1.145 | 0.000 | 0.000 | 0.000 | 0.000 |
| Alkane | Decane | 0.269 | 0.000 | 0.000 | 0.000 | 0.000 | 0.000 | 0.000 | 0.000 | 0.000 |
|  | Cyclohexane | 0.262 | 0.307 | 0.261 | 0.260 | 0.300 | 0.331 | 0.000 | 0.000 | 0.000 |
|  | Hexadecane | 0.900 | 0.232 | 0.329 | 0.000 | 1.727 | 1.536 | 0.390 | 1.807 | 0.000 |
|  | Heneicosane | 2.741 | 3.253 | 1.608 | 2.284 | 0.000 | 1.402 | 1.496 | 1.432 | 0.569 |
|  | Tetratriacontane | 0.000 | 0.406 | 0.000 | 0.345 | 0.460 | 0.000 | 0.454 | 0.447 | 0.000 |
|  | Eicosane | 0.000 | 0.000 | 0.246 | 0.000 | 1.887 | 0.000 | 2.569 | 0.000 | 0.627 |
|  | Tridecane | 0.351 | 0.274 | 0.000 | 1.434 | 1.362 | 0.000 | 0.231 | 0.209 | 0.000 |
|  | Heptadecanenitrile | 0.000 | 1.956 | 0.000 | 0.000 | 0.000 | 0.000 | 0.000 | 0.000 | 0.000 |
|  | Pentadecanenitrile | 1.539 | 0.000 | 2.263 | 2.711 | 2.834 | 1.904 | 2.570 | 2.016 | 2.653 |
|  | Tetratetracontane | 0.257 | 0.000 | 0.000 | 0.000 | 0.000 | 0.000 | 0.000 | 0.000 | 0.000 |
|  | 2-Bromo dodecane | 1.624 | 0.857 | 1.431 | 0.000 | 0.752 | 0.796 | 0.942 | 0.000 | 0.000 |
|  | 1-Pentadecyne | 0.000 | 0.613 | 0.000 | 1.103 | 1.061 | 0.894 | 0.000 | 0.000 | 0.000 |
|  | Oleanitrile | 22.623 | 3.615 | 22.221 | 23.920 | 7.270 | 5.706 | 8.271 | 6.003 | 27.089 |
|  | Octadecanenitrile | 1.190 | 1.570 | 2.047 | 2.931 | 2.333 | 1.930 | 2.379 | 1.669 | 2.945 |
|  | Heptacosane | 0.621 | 0.000 | 0.000 | 0.000 | 0.000 | 0.000 | 0.000 | 0.000 | 1.279 |
|  | 3-Heptadecene | 0.000 | 0.000 | 0.000 | 0.398 | 0.000 | 0.000 | 0.308 | 0.000 | 0.000 |
|  | Heptadecane | 0.330 | 1.363 | 0.916 | 2.608 | 1.098 | 3.382 | 0.389 | 1.154 | 0.265 |
|  | Octadecane | 0.167 | 0.000 | 0.647 | 0.000 | 0.000 | 0.000 | 0.929 | 0.327 | 0.000 |
|  | Tetracosane | 0.000 | 0.000 | 0.000 | 0.160 | 0.000 | 0.000 | 0.176 | 0.000 | 0.000 |
|  | Octacosane | 0.000 | 0.293 | 0.220 | 0.098 | 0.288 | 0.000 | 0.000 | 0.000 | 0.000 |
|  | Docosane | 0.402 | 0.000 | 0.370 | 0.000 | 0.000 | 0.299 | 0.000 | 0.000 | 0.000 |
|  | Nonadecane | 0.000 | 0.000 | 0.000 | 0.000 | 0.000 | 0.321 | 0.000 | 0.564 | 0.000 |
|  | Pentadecane | 0.000 | 0.000 | 0.000 | 0.000 | 0.000 | 0.000 | 0.737 | 0.000 | 0.000 |
|  | Dodecane | 0.000 | 0.000 | 0.000 | 0.000 | 0.000 | 0.000 | 0.000 | 0.696 | 0.000 |
|  | Undecane | 0.000 | 0.000 | 0.000 | 0.000 | 0.000 | 0.000 | 0.000 | 0.315 | 0.000 |
|  | Pentacosane | 0.000 | 0.000 | 0.000 | 0.000 | 0.000 | 0.000 | 0.000 | 2.131 | 0.483 |
|  | Hexacosane | 0.000 | 0.000 | 0.000 | 0.000 | 0.000 | 0.000 | 0.000 | 0.000 | 0.144 |
